# A revision of the genus *Eurhoptus* LeConte, 1876 (Curculionidae, Cryptorhynchinae) of America north of Mexico

**DOI:** 10.3897/zookeys.787.26948

**Published:** 2018-10-02

**Authors:** Robert S. Anderson, Michael S. Caterino

**Affiliations:** 1 Beaty Centre for Species Discovery, Research and Collection Division, Canadian Museum of Nature, PO Box 3443, Station D, Ottawa, ON. K1P 6P4, Canada Canadian Museum of Nature Ottawa Canada; 2 John and Suzanne Morse Chair of Arthropod Biodiversity, Department of Plant and Environmental Sciences, 277 Poole Agricultural Center, Clemson University, Clemson, SC 29634-0310, USA Clemson University Clemson United States of America

**Keywords:** Biodiversity, new species, phylogeny, species discovery, taxonomy, weevils

## Abstract

The genus *Eurhoptus* LeConte, 1876 is revised for America north of Mexico. Eight species are recognized including *E.pyriformis* LeConte, 1876, *E.sordidus* (LeConte, 1876), *E.curtus* (Hamilton, 1893), resurrected name, and five new species as follows: *E.rileyi* new species (type locality, Texas, Hidalgo County, Bentsen Rio Grande State Park), *E.imbricatus* new species (type locality, Texas, Bandera County, Lost Maples State Natural Area), *E.cariniventris* new species (type locality, Texas, Bandera County, Lost Maples State Natural Area), *E.occidentalis* new species (type locality, Texas, Brewster County, Big Bend National Park), and *E.aenigmaticus* new species (type locality, Alabama, Winston County, Bankhead National Forest). Descriptions or redescriptions, and images of taxonomically important structures are presented for all species. A key to the eight species is included.

## Introduction

The genus *Eurhoptus* was established by LeConte in 1876 for the species *E.pyriformis* LeConte, 1876. The genus was differentiated from the closely related *Acalles* Schoenherr, 1825 by the pear-shaped body and a stouter antennal club. Blatchley and Leng (1916) noted the presence of a deep triangular polished impression on the first ventrite in *E.pyriformis* but did not notice the similar impression in *A.sordidus* LeConte, 1876, likely because specimens are often encrusted in dirt, and left this latter species in *Acalles*. It was not until Kissinger (1964) recognized this similarity in abdominal ventrite sculpture and moved *Acallessordidus* LeConte into the genus *Eurhoptus* that the definition of the genus came to rely primarily on the presence of this variously impressed first abdominal ventrite. Until the present publication, the genus contained, in addition to these two described species from the U.S.A., nine described Mexican and Central American species (O’Brien and Wibmer 1982). Here, we add five new species and resurrect the name *A.curtus* Hamilton, 1893 as a valid species and not a synonym of *A.sordidus* LeConte, 1876. The genus is tremendously diverse from Mexico south to Panama with likely a few hundred undescribed species (Anderson and O’Brien 1996; Anderson and Ashe 2000).

At least three new species have been known to occur in the U.S. for some time (Anderson 2002), but their exact limits and relationships to the described species have remained unclear. A phylogeographic study (Caterino and Langton-Myers in press) on *E.pyriformis* revealed deep divisions within this species in the southeastern U.S., suggesting a more complex taxonomic situation than expected, and prompting the current attempt to more completely revise the taxonomy of the known and unknown U.S. species.

The natural history of *Eurhoptus* is essentially unknown. The adults are frequently sifted from various types of leaf litter, where they may feed on fungus-infested plant debris, as some related taxa are thought to do (Luna-Cozar et al. 2014). The adults are flightless, and flightless relatives in Europe have been alleged to be important indicators of well-preserved ancient woodlands (Buse 2012). This does not necessarily appear so for the North American species, which have been found in a variety of secondary forest habitats. No larvae of the genus have been found or described.

## Materials and methods

Standard taxonomic procedures for the examination of pinned specimens have been used. Maps were prepared with Simplemappr (http://www.simplemappr.net). Species identifications of outlying individuals were confirmed. GenBank numbers, label data, and voucher numbers for all sequences analyzed here are provided in Suppl. material 1: Table S1. Coordinates given for specimens examined are in decimal degrees. This study was based on 1250 specimens, obtained from (or deposited in) the following collections (listed by these acronyms in ‘Specimens examined’ sections):

ASUICArizona State University Insect Collection, Tempe, Arizona, U.S.A. (includes specimens from the Charles W. O’Brien collection)

BMNH The Natural History Museum, London, England

CMNC Canadian Museum of Nature, Ottawa, Canada

CMNH Carnegie Museum of Natural History, Pittsburgh, Pennsylvania, U.S.A.

CNCI Canadian National Collection of Insects, Ottawa, Canada

CUAC Clemson University Arthropod Collection, Clemson, South Carolina, U.S.A.

CWOB Charles W. O’Brien Insect Collection, Green Valley, Arizona, U.S.A.

EGRC Edward G. Riley Insect Collection, College Station, Texas, U.S.A.

FSCA Florida State Collection of Arthropods, Gainesville, Florida, U.S.A.

INHS Illinois Natural History Survey, Champaign, Illinois, U.S.A.

KSC Kyle Schnepp Collection, Gainesville, Florida, U.S.A.

LSAM Louisiana State Arthropod Museum, Baton Rouge, Louisiana, U.S.A.

MCZC Museum of Comparative Zoology, Cambridge, Massachusetts, U.S.A.

MEM Mississippi State Entomology Museum, Mississippi, Mississippi, U.S.A.

TAMU Texas A & M University Insect Collection, College Station, Texas, U.S.A.

TMMC University of Texas Insect Collection, Austin, Texas, U.S.A.

UAAM University of Texas Insect Collection, Austin, Texas, U.S.A.

UCFC University of Central Florida Collection of Arthropods, Orlando, Florida, U.S.A.

USNM United States National Museum, Washington D.C., U.S.A.

UTCI University of Tennessee Chattanooga Collection of Insects, Chattanooga, Tennessee, U.S.A.

### Phylogeny

In part to test the distinctiveness of the species we have recognized among Nearctic *Eurhoptus*, we undertook a molecular phylogenetic analysis including multiple representatives where possible, of as many species as possible. Our main questions regarded the distinctiveness of a few morphological variants related to both *E.pyriformis* and *E.sordidus*. Our data set largely relies on data generated for a population level analysis of *E.pyriformis* in the southern Appalachians (Caterino and Langton-Myers in press). However, we have generated a number of additional sequences for the current study, and have pruned out many redundant or highly similar sequences (mainly within *E.pyriformis*) for the present analysis. In total, the present analysis includes data from 87 *Eurhoptus* individuals, and nine outgroup Cryptorhynchinae (representatives of *Acalles*, *Peracalles*, *Trichacalles*, *Ouroporopterus*, and *Trigonopterus*, the last three from sequences previously published by Riedel et al. (2016). Thus, analyses were based on 96 taxa overall. These include representatives of four of the eight species we now recognize in *Eurhoptus*, including *E.curtus*, *E.sordidus*, *E.aenigmaticus*, and *E.pyriformis*. Extractions of pointed specimens of other species were attempted, but none succeeded. See Suppl. material 1: Table S1 for full data and GenBank accession numbers for all individuals and genes sequenced.

We dissected each specimen and used the GeneJet Genomic DNA Purification Kit (ThermoFisher Scientific, Waltham, MA) to extract DNA from the dissected head and prothorax. Following tissue digestion, we removed the remaining exoskeleton and mounted the body parts as vouchers. Most vouchers are deposited in the Clemson University Arthropod Collection. We sequenced portions of four genes for this analysis. We sequenced 826 bp of the mitochondrial cytochrome oxidase I (COI) gene, which is represented in 78 individuals. The portion sequenced is from the 5’ half of the gene, mainly using the primers C1-J-2183 (‘Jerry’) and TL2-N-3014 (‘Pat’; both from Simon et al. 1994). We developed one additional forward primer to amplify some difficult lineages, C1-J-2406 (TTYACTTCAGCWACWATAATYATTGC). Three nuclear genes were targeted, mainly selecting one or two individuals for each distinct mitochondrial haplotype. These included partial sequences of the internal transcribed spacer 2 (ITS2) for 56 individuals, using the primers TW81 and HITR (from Richards et al. 1997), aligned length 1611 bp; 921 bp of the protein coding rudimentary (CAD) gene from 78 individuals, using the primers CD821F and CD1098R2 (from Wild and Maddison 2008); and 344 bp of an intron in the *krotzkopf verkehrt* (kkv) gene from 67 individuals using the primers KKV2768F and KKV3023R (from Polihronakis-Richmond and Caterino 2012). Amplifications via PCR started with a 3-minute initial denaturation at 95°, a 30 second denaturation at 94°, 35–40 cycles with annealing temperatures of 50–62° (see Suppl. material 1: Table S1 for details), a 1-minute extension at 72°, and a 5-minute final extension at 72°. Successful PCR products were sent to Macrogen USA (Rockville, MD) for Sanger sequencing in both directions. Sequence chromatograms were compiled, inspected, and preliminarily aligned in Geneious (Auckland, NZ).

Sequences of COI were length invariant, and alignment was trivial. Sequences of all other markers included length variation. These were aligned using MAFFT online (Katoh et al. 2017), using the ‘Auto’ setting, which selected an internal algorithm depending on number, length, and complexity of sequences. Combined sequences were analyzed under parsimony in PAUP* (Swofford 2002), with all sequences weighted equally, gaps treated as missing data, 100 random sequence addition replicates, saving no more than 1000 trees at each step; and under Bayesian criteria using MrBayes (v.3.2.6 [Ronquist and Huelsenbeck 2003]) on the CIPRES Science Gateway, partitioning the four-gene data set, and implementing a GTR+I+Γ model; nruns=2, ngen=10,000,000, nchains=4, burninfrac=0.25.

## Results and discussion

### Phylogeny

There is a high degree of molecular diversity and divergence among species and populations of Nearctic *Eurhoptus*. Uncorrected pairwise divergences in COI exceeded 10% between all of the species we recognize below (for which we had data). Some still contain intraspecific divergences approaching this. However, the higher divergences in these cases are spanned by intervening populations, and are united by morphology, and we do not yet see clear grounds for further separation.

**Figure 1.**
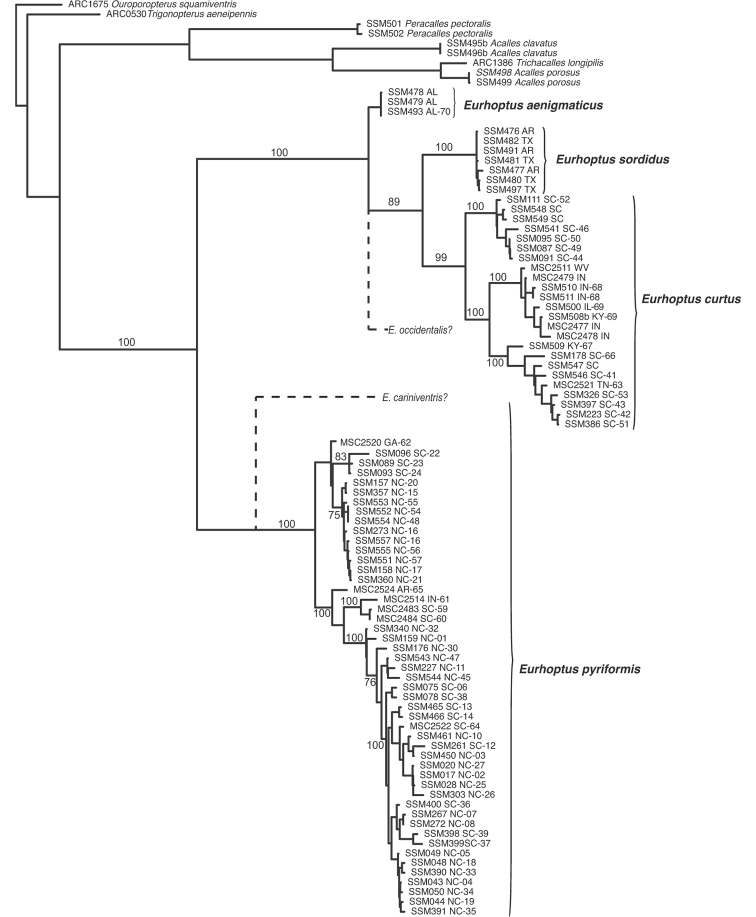
One of a number of equally parsimonious trees (numerous rearrangments within species were equally parsimonious). Numbers on branches represent Bayesian posterior probabilities >75% for major clades. Terminal units are named by DNA extraction code/voucher number, followed by state of collection (two letter abbreviation) and COI haplotype, as reported in Caterino and Langton-Myers (in press), or newly here. Dotted lines represent hypothetical placements of two other species, yet unsampled for DNA.

**Figure 2.**
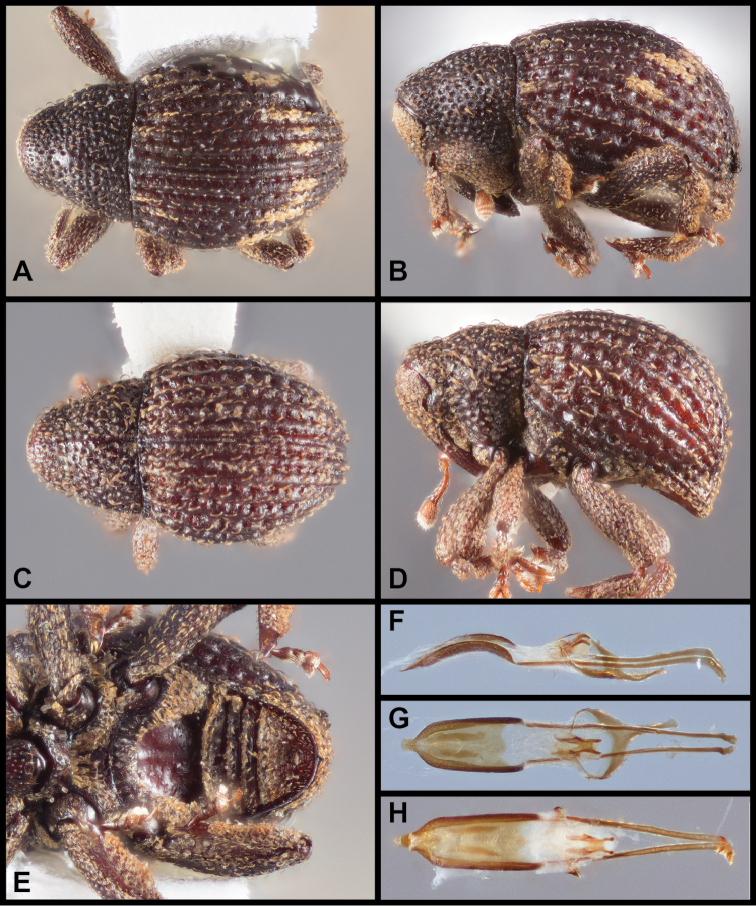
*Eurhoptuspyriformis*. **A** Dorsal view of scaled form **B** Lateral view of scaled form **C** Dorsal view of ‘plain’ form **D** Lateral view of ‘plain’ form **E** Ventral view showing impressions on ventrites **F** Lateral view of aedeagus **G, H** Dorsal views showing range of variation of aedeagus.

Both parsimony and Bayesian analyses (see Figure 1) supported a deep primary division between *E.pyriformis* and three species very similar to and including *E.sordidus*, which exhibit a narrowed pronotal base. Preliminary morphological work suggested that ‘*E.pyriformis*’, as previously circumscribed (all those populations with a broad pronotal base), contained up to three species. One of these, referred to below as *E.cariniventris* n. sp., was not represented by molecular data, but appears justifiably distinct based on male genitalia. Another morphotype, referred to in Caterino and Langton-Myers (in press) as ‘patterned *E.pyriformis*’ turns out to be neither genetically nor morphologically supportable. Individuals showing these distinctively patterned elytra (Figures 2A, B) are now known from across the range (specifically individuals from NC, GA, and AR), and these are resolved as paraphyletic with respect to a ‘plain’ morphotype. Genitalia are somewhat variable in both, particularly with regard to the shape of the aedeagal tip.

Morphological and molecular data agree in distinguishing multiple species within what has been referred to as *E.sordidus*. *Eurhoptussordidus* itself is restricted to eastern Texas, western Arkansas, and eastern Oklahoma. Sequenced individuals from Arkansas and Texas are strongly supported as monophyletic, and minimally divergent among themselves. A largely more eastern lineage is strongly supported as the sister to *E.sordidus*, and for this we resurrect Hamilton’s (1893) name *E.curtus*. There is some potentially interesting subdivision among *E.curtus*, particularly with regard to some southeastern-most representatives (South Carolina specimens from Greenville and McCormick Counties). However, molecular sampling across this widespread species’ broad range is not yet adequate to assess variation patterns. A third species in the *sordidus* lineage is that which we describe below as *E.aenigmaticus*. This is restricted to a rather small area of southern Alabama, Mississippi, and Lousiana. The genitalia of this species is quite distinct. We’ve only been able to sequence DNA from Alabama specimens, and their monophyly is not well supported (probably because COI has only been sequenced for two of the four individuals). However, it appears to represent a sister to *E.sordidus* + *E.curtus*.

With regard to species not represented by molecular data, we would predict that *E.cariniventris* would be resolved as the sister to *E.pyriformis*. *Eurhoptusoccidentalis* from west Texas appears morphologically close to *E.sordidus*, and would likely fall out somewhere in that clade. *Eurhoptusimbricatus* and *E.rileyi* would appear further from these other Nearctic species, and may represent one or more independent lineages with closer relationships to groups known from Mexico and southward. Much broader representation from this largely undescribed Neotropical fauna will be necessary before anything can be said about their relationships.

### Taxonomy

#### Eurhoptus

Taxon classificationAnimaliaColeopteraCurculionidae

LeConte, 1876

Eurhoptus LeConte, 1876: 245; Blatchley and Leng 1916: 496; Kissinger 1964: 64; Papp 1979: 163; O’Brien and Wibmer 1982: 136; Downie and Arnett 1996: 1581; Alonso-Zarazaga and Lyal 1999: 131; Salsbury 2000: 347; Anderson 2002: 765; Ciegler 2010: 166.
Europtus ; Fiedler 1940: 300 (error, in key).
Eurrhoptus Rye 1878: 93 (unjustified emendation).

##### Type species.

*Eurhoptuspyriformis* LeConte, 1876: 245 (by monotypy). Gender masculine.

##### Redescription

(U.S.A. species only). Small, convex, rounded, dull black or dark-brown. Body length (exclusive of head and rostrum) 1.8–3.2 mm, cuticle either largely bare, variously covered with short, fine to coarse, recurved seta-like scales, lacking other scales or variously clothed with dense approximate to imbricate flat scales in addition to recurved seta-like scales. Rostrum short, stout, flattened dorsally, about as long as pronotum or slightly less, scaly towards base, glabrous, finely punctured towards apex in female, more coarsely so in male; medially carinate or not. Eyes small, flat, oval, largely covered by slight post-ocular lobes when rostrum in repose. Head with frons scaly, not impressed. Antennae red-brown, funicle of 7 desmomeres, club small, oval. Pronotum about as wide as long, lateral margins rounded or straight; if straight, then margins tapered more or less evenly from base to apex with greatest width at base; if rounded, then apical portion constricted, tubulate with greatest width before base. Basal margin nearly straight, disc variously punctured, medially carinate, sulcate or evenly punctured; post-ocular lobes slight. Elytra robust, strongly rounded dorsally and laterally, striae distinct, of small to very large punctures; all elytral intervals evenly elevated, slightly rounded, each with single row of recurved seta-like scales. Scutellar shield not visible. Mesoventral cup distinct, metaventrite short, medially impressed behind cup or carinate. Abdomen with ventrite 1 variously modified with large median or paired rather deep pits, pit either glabrous, shining or filled with erect scales; areas around pit often with dense, fine golden setae encircling depression; ventrite 5 broadly triangular, about as long as ventrites 2–4 combined. Legs with femora not toothed, narrow to stout (greatly so in some species where width accentuated by dense, erect scales along dorsal and lateral margins); tibiae narrow to stout (greatly so in some species where width accentuated by dense, erect scales along dorsal and lateral margins); tarsi fine, narrow, article 3 widest, bilobed; tarsal claws minute. Male with aedeagus short, one-half or less length of aedeagal apodemes, in lateral view very slightly curved ventrally to almost straight, in dorsal view with lateral margins subparallel, slightly to abruptly convergent towards apex at about apical one-third to one-fifth. Internal sac with apical sclerite complex of modified cruciform arrangement. Female with bursa copulatrix and vagina lacking any internal sclerotization, spermatheca L-shaped; distal gonocoxite elongate-triangular, with distinct long, slender apical stylus, sternite 8 with apical lamellae short, basally widely divergent, basal apodeme rather robust, elongate, expanded slightly at apex, tergite 8 tapered towards truncate or acuminate finely irregularly serrate apex.

##### Diversity.

For many years two species have been recorded as occurring in the eastern U.S.A., *E.pyriformis* and *E.sordidus*; however, specimens from central Texas of *E.sordidus* differed in the structure of ventrite 1 with two separate impressions and not a single larger impression thus suggesting a third species was present. This species and two additional undescribed species were noted by Anderson (2002).

##### Natural history.

Almost all records of specimens of *Eurhoptus* are from sifting leaf litter in various forested habitats. Otherwise, nothing is known of life history and immature stages.

#### Eurhoptus pyriformis

Taxon classificationAnimaliaColeopteraCurculionidae

LeConte, 1876

Figures 2 , 3

Eurhoptus pyriformis LeConte, 1876: 245; Salsbury 2000: 347; Ciegler 2010: 167.
Eurhoptus pyriformis (part); Blatchley and Leng 1916: 497; Kissinger 1964: 64; Papp 1979: 164; O’Brien and Wibmer 1982: 136; Downie and Arnett 1996: 1581; Alonso-Zarazaga and Lyal 1999: 131; Anderson 2002: 765.

##### Notes about types.

This species was described from a single specimen labelled “Ill[inois]”. It has a red square label with “Type 5316” and a handwritten label “Eurhoptuspyriformis Lec.”. It is not dissected but appears to be a male based on the more coarsely punctate rostral apex. The type was not examined although high resolution images posted on the MCZC Online Type Database (http://140.247.96.247/mcz/Species_record.php?id=5050) were examined. There is little doubt as to the identity of this species.

##### Redescription.

Body length (exclusive of head and rostrum) 2.1–2.9 mm, cuticle largely bare, variously covered with short, fine recurved seta-like scales, most specimens lacking other scales but some with head and base of rostrum scaly and with a few broad, flat pale imbricate scales variously arranged in irregular oblique band across elytra in apical one-half on intervals 2–7 and at bases of intervals 3–4 and 7. Pronotum with lateral margins straight, margins tapered more or less evenly from base to apex with greatest width at base, not medially carinate but may be medially impunctate in some specimens. Elytra strongly rounded dorsally and laterally, striae distinct, of large punctures, especially in posthumeral region; all elytral intervals evenly elevated, slightly rounded, each with single row of fine recurved seta-like scales. Metaventrite medially deeply impressed behind mesoventral cup. Abdomen with ventrite 1 with large median deep glabrous shining pit, area around pit with dense, fine golden inwardly directed scales encircling depression. Legs with femora and tibiae at most fringed with a few erect scales especially along outer margin of tibiae, hind tibiae subequal in width throughout most of length. Aedeagus about as long as one-half length of aedeagal apodemes, in lateral view slightly ventrally curved, in dorsal view with margins subparallel but abruptly strongly tapered towards apex at about apical one-quarter to one-fifth, apex produced medially as narrow extension, broadly rounded at tip, extension slightly narrower or more acuminate in some (scaly) specimens. Internal sac with large distinct cruciform apical sclerite complex, anterior bars longer than posterior bars, bars well defined, subparallel.

##### Distribution

**Figure 3.**
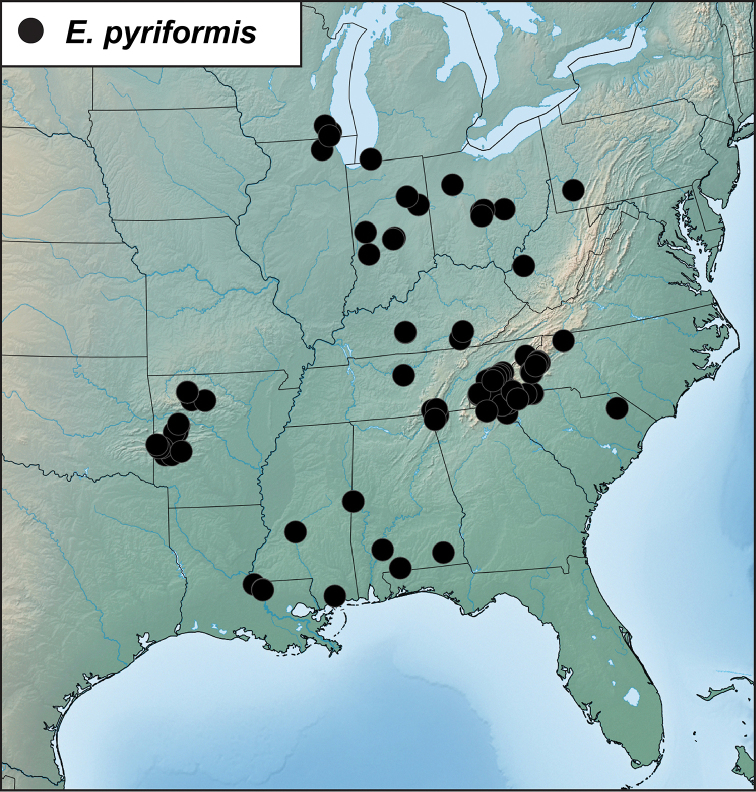
Records of *Eurhoptuspyriformis*.

(Figure 3). This species is widely distributed in the eastern U.S.A. from Illinois, Pennsylvania and Michigan in the north, to the Gulf Coast of Mississippi and Louisiana in the south, and from Arkansas in the west, east to the Carolinas. Any literature records of this species from Texas are of *E.cariniventris*. Salsbury (2000) records a single specimen of this species from Kansas without further details; Ciegler (2010) records it from South Carolina. Blatchley and Leng (1916), followed by O’Brien and Wibmer (1982) include Colorado in the distribution but this seems in error.

##### Notes about variation.

Some specimens examined have a few broad, flat pale imbricate scales variously arranged in an irregular oblique band across the elytra in the apical one-half on intervals 2–7 and at bases of intervals 3–4 and 7 (Figures 2A–B). These specimens also possess a slightly narrowed apex of the aedeagus but otherwise do not seem to be different from unscaled specimens and the two forms appear to be broadly sympatric. Sequences available to date are predominantly from southern Appalachia, the majority of which represent the unscaled form (Figures 2C–D). However, the scaled form is in fact more widespread, ranging from Arkansas in the west to South Carolina, and north to Michigan, and the molecular data suggest that this form is paraphyletic with respect to those unscaled populations in Appalachia. Similar scaling and the structure of abdominal ventrite 1 in *E.cariniventris* lead us to suggest its relationship as sister to *E.pyriformis*.

##### Specimens examined

(293). **ALABAMA**: Escambia Co.: Brewton, Jct. Hwy 41 & Escambia R. Trib (31.07, -87.06), C.W. O’Brien & P.W. Kovarik, 31 Dec 1997, leaf litter from beech-magnolia forest (1, ASUIC); Monroe Co.: 1.6 km. S Claiborne Dam (31.5917, -87.5392), C.E. Carlton, 31 May 1995, beech/magnolia riparian berlese (1, ASUIC); Sumter Co.: Highway 17 at Noxubee River (32.918, -88.298), J.A. McGown, 27 May 2004, under dead *Quercusnigra*, floodplain (1, MEM). **ARKANSAS**: Logan Co.: Mount Magazine, 0.64 km. S. Green Field Park (35.167, -93.645), C. Carlton, 21 May 1987, (4, CMNC); Logan Co.: Mount Magazine, Green Bench, Gutter Rock Ck. (35.21, -93.66), D. Bowles, 5 May 1988, leaf litter (1, UAAM); Logan Co.: Mount Magazine, Signal Hill Trail (35.17, -93.64), E.G. Riley, 13 May 1995 (6, EGRC); Montgomery Co.: 2.41 km. E. Crystal Recreation Area, slopes at FS 177K (34.5, -93.6), C.E. Carlton & H.W. Robison, 19 Dec 1991, deciduous litter (1, UAAM); Montgomery Co.: 2.41 km. E. Crystal Recreation Area, slopes at FS 177K (34.5, -93.6), C.E. Carlton & H.W. Robison, 24 Mar 1992, deciduous litter (1, UAAM); Montgomery Co.: 2.41 km. E. Crystal Recreation Area, slopes at FS 177K (34.5, -93.6), C.E. Carlton & H.W. Robison, 4 May 1992, deciduous litter (1, UAAM); Montgomery Co.: 2.41 km. E. Crystal Recreation Area, slopes at FS 177K (34.5, -93.6), C.E. Carlton & H.W. Robison, 16 May 1992, deciduous litter (1, UAAM); Montgomery Co.: 2.41 km. E. Crystal Recreation Area, slopes at FS 177K (34.5, -93.6), C.E. Carlton & H.W. Robison, 16 Nov 1991, deciduous litter (1, UAAM); Montgomery Co.: Collier Spring (34.4839, -93.5929), C.E. Carlton & J.S. Park, 17 Oct 2012, berlese (2, CUAC), DNA extract SSM431; Montgomery Co.: Crystal Recreation Area, E. of campground (34.5, -93.6), C.E. Carlton & H.W. Robison, 21 Jan 1992, deciduous litter (1, UAAM); Montgomery Co.: Little Missouri Falls Recreation Area, slopes SW of river (34.4, -93.9), C.E. Carlton & H.W. Robison, 17 Aug 1991, deciduous litter (1, UAAM); Montgomery Co.: Little Missouri Falls Recreation Area, slopes SW of river (34.4, -93.9), C.E. Carlton & H.W. Robison, 26 Jul 1991, deciduous litter (1, UAAM); Newton Co.: Buffalo National River (36.0369, -93.3411), M.J. Skvarla, 12 Jun 2013, litter next to log (1, UAAM); Newton Co.: Buffalo National River (36.0369, -93.3411), M.J. Skvarla, 25 Sep 2013, litter next to log (1, UAAM); Newton Co.: Buffalo National River (36.0372, -93.3408), M.J. Skvarla, 25 Sep 2013, leaf litter in pile (1, UAAM); Newton Co.: Buffalo National River (36.0372, -93.3411), M.J. Skvarla, 25 Sep 2013, thin litter next to log (1, UAAM); Newton Co.: Buffalo National River (36.03777, -93.34), M.J. Skvarla, 29 May 2013, pitfall in wooded area (1, UAAM); Newton Co.: Buffalo National River (36.0386, -93.3403), M.J. Skvarla, 28 Aug 2013, pitfall (1, UAAM); Newton Co.: Buffalo National River (36.0386, -93.3411), M.J. Skvarla, 28 Jun 2013, red pan/intercept (1, UAAM); Newton Co.: Buffalo National River (36.0386, -93.34), M.J. Skvarla, 29 May 2013, pitfall in wooded area (1, UAAM); Newton Co.: Buffalo National River (36.0389, -93.3408), M.J. Skvarla, 17 Jul 2013, litter under low bush (1, UAAM); Newton Co.: Buffalo National River (36.0389, -93.34), M.J. Skvarla, 29 May 2013, pitfall in wooded area (1, UAAM); Newton Co.: Natural Bridge at Deer (35.827, -93.21), R.T. Allen, 1 Jun 1987, (1, CMNC); Polk Co.: Caney Creek Wilderness Area, 5.63 km. N. Bard Springs (34.4, -94.1), C.E. Carlton & H.W. Robison, 18 Dec 1991, maple beech litter (1, UAAM); Polk Co.: Caney Creek Wilderness Area, 5.63 km. N. Bard Springs (34.4, -94.1), C.E. Carlton & H.W. Robison, 6 Jul 1991, maple beech litter (1, UAAM); Polk Co.: Caney Creek Wilderness Area, 5.63 km. N. Bard Springs (34.4, -94.1), C.E. Carlton & H.W. Robison, 7 Mar 1992, maple beech litter (1, UAAM); Polk Co.: Caney Creek Wilderness Area, 5.63 km. N. Bard Springs (34.4, -94.1), C.E. Carlton & H.W. Robison, 24 Mar 1992, maple beech litter (1, UAAM); Polk Co.: Caney Creek Wilderness Area, 5.63 km. N. Bard Springs (34.4, -94.1), C.E. Carlton & H.W. Robison, 15 Nov 1991, maple beech litter (1, UAAM); Polk Co.: Caney Creek Wilderness Area, 5.63 km. N. Bard Springs (34.4, -94.1), C.E. Carlton & H.W. Robison, 28 Sep 1991, maple beech litter (1, UAAM); Polk Co.: Rich Mt., Acorn Overlook (34.6, -94.2), C.E. Carlton & H.W. Robison, 5 Jun 1992, deciduous litter (1, UAAM); Polk Co.: Rich Mt., Eagleton Overlook (34.6, -94.3), C.E. Carlton & H.W. Robison, 9 Jan 1992, deciduous litter (1, UAAM); Polk Co.: Rich Mt., intersec 272-88 (34.68, -94.36), C.E. Carlton & H.W. Robison, 25 Jul 1991, deciduous litter (1, UAAM); Polk Co.: Rocky, Talimena Drive (34.619, -94.276), D. Beyers; M. Jenks, 14 Apr 1984, hardwood berlese (5, CMNC); Scott Co.: Dry Creek Wilderness Area (35, -93.7), C.E. Carlton & H.W. Robison, 15 May 1992, deciduous litter (1, UAAM); Scott Co.: Hogan Mt. Turkey Area (35, -93.8), C.E. Carlton & H.W. Robison, 25 Aug 1991, deciduous litter (1, UAAM); Searcy Co.: 6 mi N Co line on Hwy 27 (35.8, -92.78), C.E. Carlton & H.W. Robison, 30 Aug 1977, deciduous litter (1, UAAM); Yell Co.: 6.44 km. S. Blue Mt. Lake (35, -93.7), C.E. Carlton & H.W. Robison, 18 Jun 1992, deciduous litter (1, UAAM). **GEORGIA**: Dade Co.: Cloudland Canyon State Park, (34.83, -85.48), W. Suter, 21 Apr 1983, rhododendron litter (1, ASUIC); Dade Co.: Cloudland Canyon State Park, (34.83, -85.48), S. & J. Peck, 16 May 1972, litter (3, CNCI); Rabun Co.: Rabun Bald (34.97, -83.3), Q. Wheeler, 2 Aug 1981, leaf litter (1, ASUIC); Rabun Co.: Rabun Bald (34.97, -83.3), Q. Wheeler, 11 Aug 1981, leaf litter (1, ASUIC); Rabun Co.: Summit (34.97, -83.3), Q. Wheeler, 5 Jun 1981, leaf litter (1, ASUIC); Union Co.: 9.66 km. E. Blairsville, Brasstown Bald (34.87, -83.81), W. Suter, 15 Jun 1973, Rhododendron litter (1, CWOB); Walker Co.: Lula Lake (34.9312, -85.3733), S. Chatzimanolis, 18 May 2015, sifted litter (1, UTCI); Walker Co.: Lula Lake (34.9315, -85.3729), S. Chatzimanolis, 1 Dec 2012, sifted litter (1, UTCI); Walker Co.: Lula Lake (34.9338, -85.3716), S. Chatzimanolis, 26 May 2015, sifted litter (1, UTCI), DNA extract MSC2520. **ILLINOIS**: McHenry Co.: Algonquin (42.2, -88.3), W. Nason, (1, INHS). **INDIANA**: Huntington Co.: 3.22 km. E. Mount Etna (40.74, -85.51), R. Wilkey, 5 May 1977 (2, CWOB); Marion Co.: Southport (39.7, -86.1), R.F. Wilkey, 28 Apr 1978, (1, ASUIC); Marion Co.: 8.05 km. S. Indianapolis (39.67, -86.15), 10 Jul 1977, maple/Celtis litter (2, CWOB); Owen Co.: 8.05 km. N.W. Clay City (39.34, -87.03), R. Wilkey, 17 Aug 1973, berlese duff (2, CWOB); Tippecanoe Co.: 22 May 1971, N. Downie (1, CWOB); Montgomery Co.: Shades State Park, Pine Hills (39.93, -87.07), J. Wagner, 23 Aug 1960, beech tree hole (1, CWOB); Wells Co.: Bluffton 4H Park (40.897, -83.893), R. Wilkey, 25 Apr 1973, berlese *Fagus* duff (1, CMNC; 5, CWOB); Wells Co.: 8.05 km. S. Bluffton (40.68, -85.16), R. Wilkey, 24 Sep 1974 (3, CWOB). **KENTUCKY**: Edmonson Co.: Mammoth Cave National Park, Cave Entrance Trail (37.185, -86.099), R.S. Anderson, 24 Jun 1988, berlese hardwood litter (1, CMNC); Edmonson Co.: Mammoth Cave National Park, Cabin Woods (37.20, -86.10), W. Suter, 29 Mar 1974, litter (1, CWOB); Laurel Co.: Bald Rock Picnic Area (37.03, -84.22), C.E. Carlton, 4 Apr 1991, hardwood berlese (1, ASUIC); Whitley Co.: 1.6 km. E. Cumberland Falls. Road 195 (36.838, -84.344), S. O’Keefe, 22 Aug 1990, (1, CMNC). **LOUISIANA**: West Feliciana Par.: Tunica Hills Wildlife Management Area, Magnolia Glen Trail (30.94, -91.51), D. Pashley, 15 Jul 1995 (2, LSAM). West Feliciana Par.: nr. St. Francisville, Tunica Hills Wildlife Management Area (30.94, -91.51), S. Dash, 23 Sep 1999, lower ravine berlese (1, CWOB). West Feliciana Par.: nr. St. Francisville, Tunica Hills Wildlife Management Area (30.94, -91.51), J. & T. Fassbender, 23 Sep 1999, lower ravine berlese (1, CWOB); West Feliciana Par.: Feliciana Preserve (30.78, -91.25), J. Fassbender, 14 Jul 1998, mixed pine/hardwood litter (2, LSAM);. West Feliciana Par.: D.P. Cabin (30.795, -91.256), J.L. Johnson, 21 Dec 1995, berlese (2, CWOB); **MICHIGAN**: Berrien Co.: Warren Woods State Park (41.83, -86.62), D.S. Chandler, 19 Aug 1990, beech log litter (1, ASUIC). **MISSISSIPPI**: Hinds Co.: Lefleur’s Bluff State Park (32.24, -90.15), J.A. MacGown, 20 Jul 2011, berlese bottomland hardwood forest (1, MEM); Harrison Co.: 3 mi NW Ridgeway (30.5, -89.1), D.S. Chandler, 27 Oct 1978, litter (1, ASUIC). **NORTH CAROLINA**: Alleghany Co.: Roaring Gap, 2000’, Stone Mountain State Park (36.382, -81.022), S.B. Peck, 16 Aug 1981, log - leaf litter (1, CMNC); Avery Co.: Linville Falls, 3500’, Blue Ridge Parkway km. 317 (35.959, -81.942), S.B. Peck, 16 Aug 1981, Rhodendron litter at log (2, CMNC); Burke Co.: Linville Gorge Wilderness (35.922, -81.9175), S.S. Myers & L. Vasquez-Velez, 18 Mar 2016, sifted litter (1, CUAC), DNA extract SSM567b; Burke Co.: Linville Gorge Wilderness (35.9241, -81.9131), S.S. Myers & L.Vasquez-Velez, 18 Mar 2016, sifted litter (1, CUAC), DNA extract SSM556b; Burke Co.: Linville Gorge Wilderness (35.9294, -81.9109), S.S. Myers & L. Vasquez-Velez, 18 Mar 2016, sifted litter (1, CUAC), DNA extract SSM365; Burke Co.: Linville Gorge Wilderness, Bynum Bluff Trail (35.9331, -81.9282), M.S. Caterino & P.R. Caterino, 19 Aug 2017, sifted litter (4, CUAC), DNA extract SSM555; Burke Co.: Linville Gorge Wilderness, Pine Gap Trail (35.9403, -81.9297), M.S. Caterino & P.R. Caterino, 19 Aug 2017, sifted litter (5, CUAC), DNA extract SSM551; Cherokee Co.: Nantahala National Forest, Hickory Branch Trail (35.2118, -83.6987), S.S. Myers, 26 Jul 2015, sifted hardwood litter (1, CUAC), DNA extract SSM335; Cherokee Co.: Nantahala National Forest, Hickory Branch Trail (35.2165, -83.7047), S.S. Myers, 26 Jul 2015, sifted hardwood litter (2, CUAC), DNA extracts SSM240-SSM241; Cherokee Co.: Nantahala National Forest, Hickory Branch Trail (35.2176, -83.7055), S.S. Myers, 26 Jul 2015, sifted hardwood litter (6, CUAC), DNA extracts SSM043-SSM046, SSM327-SSM328; Cherokee Co.: Nantahala National Forest, London Bald Trail (35.2179, -83.7062), S.S. Myers, 26 Jul 2015, sifted hardwood litter (4, CUAC), DNA extracts SSM047-SSM050; Cherokee Co.: Nantahala National Forest, London Bald Trail (35.2231, -83.7011), S.S. Myers, 26 Jul 2015, sifted hardwood litter (6, CUAC), DNA extracts SSM331-SSM332, SSM389-SSM392; Graham Co.: Joyce Kilmer Memorial Forest (35.3426, -83.966), M.S. Caterino & S.S. Myers, 24 Jun 2015, sifted litter (2, CUAC), DNA extracts SSM028, SSM305; Graham Co.: Joyce Kilmer Memorial Forest (35.3433, -83.9621), S.S. Myers, 20 Jul 2015, sifted litter (2, CUAC), DNA extracts SSM017, SSM030; Graham Co.: Joyce Kilmer Memorial Forest (35.3433, -83.966), M.S. Caterino & S.S. Myers, 24 Jun 2015, sifted litter (2, CUAC), DNA extracts SSM026, SSM304; Graham Co.: Joyce Kilmer Memorial Forest (35.3435, -83.977), S.S. Myers, 20 Jul 2015, sifted litter (1, CUAC), DNA extract SSM020; Graham Co.: Joyce Kilmer Memorial Forest (35.3441, -83.966), M.S. Caterino & S.S. Myers, 24 Jun 2015, sifted litter (2, CUAC), DNA extracts SSM027, SSM303; Graham Co.: Joyce Kilmer Memorial Forest (35.3448, -83.9649), M.S. Caterino & S.S. Myers, 24 Jun 2015, sifted litter (2, CUAC), DNA extracts SSM029, SSM306; Graham Co.: Joyce Kilmer Memorial Forest (35.345, -83.967), M.S. Caterino & S.S. Myers, 24 Jun 2015, sifted litter (2, CUAC), DNA extracts SSM017, SSM025; Graham Co.: Joyce Kilmer Memorial Forest (35.3467, -83.9688), S.S. Myers, 20 Jul 2015, sifted litter (1, CUAC), DNA extract SSM019; Graham Co.: (35.32, -83.9918), S. Chatzimanolis, 12 Sep 2014, sifted litter (1, UTCI); Haywood Co.: Great Smoky Mountains National Park (35.816, -83.121), J.A. MacGown; J.G. Hill, 11 Jun 2009, rock pine/hardwood forest (1, MEM); Haywood Co.: Great Smoky Mountains National Park, Balsam Mountain Trail, 1500 m (35.637, -83.183), A. Tishechkin, 20 Oct 2001, hardwood berlese (1, CMNC); Jackson Co.: Balsam Mountain Preserve (35.3772, -83.0921), S.S. Myers, 17 Jun 2015, sifted litter (3, CUAC), DNA extracts SSM542-SSM544; Jackson Co.: Balsam Mountain Preserve (35.398, -83.1088), S.S. Myers, 17 Jun 2015, sifted rich cove litter (2, CUAC), DNA extracts SSM224, SSM227; Jackson Co.: Nantahala National Forest, Bad Creek Trail (35.002, -83.1071), S.S. Myers, 29 Jun 2015, sifted litter (5, CUAC), DNA extracts SSM450-SSM454; Macon Co.: Highlands (35.05, -83.19), Q. Wheeler, 4 Jun 1981, leaf litter (3, ASUIC); Macon Co.: Highlands, California Gap (35.1, -83.29), Q. Wheeler, 12 Aug 1981, leaf litter (1, ASUIC); Macon Co.: Highlands, Cows Bald (35.05, -83.19), Q. Wheeler, 9 Jun 1981, leaf litter (1, ASUIC); Macon Co.: Highlands, Franklin (35.2, -83.4), Q. Wheeler, 8 Jun 1981, leaf litter (1, ASUIC); Macon Co.: Highlands, Franklin (35.2, -83.4), Q. Wheeler, 10 Jun 1981, leaf litter (1, ASUIC); Macon Co.: Nantahala National Forest, Ellicott Rock Trail (35.0075, -83.1348), S.S. Myers, 18 Jul 2015, sifted litter (2, CUAC), DNA extracts SSM253-SSM254; Macon Co.: Nantahala National Forest, Ellicott Rock Trail (35.0096, -83.1245), S.S. Myers, 18 Jul 2015, sifted litter (6, CUAC), DNA extract SSM255-SSM260; Macon Co.: Nantahala National Forest, Hickory Gap (35.0697, -83.2825), S.S. Myers, 16 Jul 2015, sifted litter (2, CUAC), DNA extracts SSM456-SSM457; Macon Co.: Nantahala National Forest, Hickory Gap (35.0746, -83.2855), S.S. Myers, 16 Jul 2015, sifted litter (3, CUAC), DNA extracts SSM342-SSM343, SSM455; Macon Co.: Nantahala National Forest, Highlands (35.05, -83.19), Q. Wheeler, 4 Jun 1981, leaf litter (1, ASUIC); Macon Co.: Nantahala National Forest, Highlands (35.05, -83.19), Q. Wheeler, 6 Jun 1981, leaf litter (1, ASUIC); Macon Co.: Nantahala National Forest, Highlands (35.05, -83.19), Q. Wheeler, 7 Jun 1981, leaf litter (1, ASUIC); Macon Co.: Nantahala National Forest, Highlands (35.05, -83.19), Q. Wheeler, 8 Jun 1981, leaf litter (1, ASUIC); Macon Co.: Nantahala National Forest, Jones Gap (35.0752, -83.2883), S.S. Myers, 16 Jul 2015, sifted litter (2, CUAC), DNA extracts SSM340-SSM341; Macon Co.: Nantahala National Forest, Jones Gap (35.0785, -83.2923), S.S. Myers, 22 Jul 2015, sifted litter (2, CUAC), DNA extracts SSM176-SSM177; Macon Co.: Nantahala National Forest, Jones Gap (35.0801, -83.2947), S.S. Myers, 22 Jul 2015, sifted litter (3, CUAC), DNA extracts SSM169-SSM172; Macon Co.: Nantahala National Forest, Jones Gap (35.0841, -83.2976), S.S. Myers, 28 Jul 2015, sifted litter (8, CUAC), DNA extracts SSM159-SSM162, SSM393-SSM396; Macon Co.: Standing Indian Campground (35.0761, -85.5284), S. Chatzimanolis, 18 Oct 2014, sifted litter (1, UTCI); McDowell Co.: Blue Ridge Parkway, mile 320 (35.94, -81.96), Q. Wheeler, 4 Aug 1981, leaf litter (1, ASUIC); McDowell Co.: Mackey Mountain Trail (35.7224, -82.1725), S.S. Myers & S. Langton, 25 Jul 2015, sifted litter (4, CUAC), DNA extract SSM274; McDowell Co.: Mackey Mountain Trail (35.7246, -82.1838), S.S. Myers & S. Langton, 25 Jul 2015, sifted litter (1, CUAC), DNA extract SSM273; McDowell Co.: Pisgah National Forest, Courthouse Falls Trail (35.2716, -82.8964), S.S. Myers, 23 Jul 2015, sifted litter (1, CUAC), DNA Extract SSM271; McDowell Co.: Pisgah National Forest, Courthouse Falls Trail (35.2727, -82.8932), S.S. Myers, 23 Jul 2015, sifted litter (1, CUAC), DNA Extracts SSM267-SSM270; McDowell Co.: Pisgah National Forest, Courthouse Falls Trail (35.2747, -82.8902), S.S. Myers, 23 Jul 2015, sifted litter (1, CUAC), DNA Extracts SSM272, SSM266; McDowell Co.: Snooks Nose (35.6905, -82.2055), S.S. Myers, 17 Mar 2016, sifted litter (3, CUAC), DNA extracts SSM320; McDowell Co.: Snooks Nose (35.6928, -82.207), S.S. Myers, 17 Mar 2016, sifted litter (4, CUAC), DNA extracts SSM357; McDowell Co.: Snooks Nose (35.693, -82.2061), S.S. Myers & L. Vasquez-Velez, 17 Mar 2016, sifted litter (1, CUAC), DNA extract SSM361; Mitchell Co.: Blue Ridge Parkway, mile 329 (35.86, -82.04), Q. Wheeler, 4 Aug 1981, leaf litter (1, ASUIC); Sevier Co.: Great Smoky Mountains National Park, Chimneys Picnic Area Nature Trail, 891 m (35.635, -83.496), A. Tishechkin; V. Moseley, 28 Jun 2001, leaf litter berlese (1, CMNC); Swain Co.: Great Smoky Mountains National Park, Flat Creek Trail, 1500 m (35.55, -83.172), A. Tishechkin, 31 Jul 2001, leaf litter berlese (1, CMNC); Swain Co.: off Coopers Creek Rd. (35.4825, -83.3797), M. Caterino, 15 Mar 2015, sifted litter (1, CUAC), DNA extract SSM470; Swain Co.: off Coopers Creek Rd. (35.4825, -83.378), M. Caterino, 15 Mar 2015, sifted riparian litter (2, CUAC), DNA extracts SSM468-SSM469; Transylvania Co.: Pisgah National Forest, off Blue Ridge Parkway (35.2871, -82.908), S.S. Myers, 29 May 2015, sifted litter (2, CUAC), DNA Extracts SSM458-SSM459; Transylvania Co.: Pisgah National Forest, off Blue Ridge Parkway (35.2915, -82.9136), S.S. Myers, 29 May 2015, sifted litter (2, CUAC), DNA Extracts SSM459-SSM460; Yancey Co.: Mount Mitchell Viewpoint, 1451 m (35.723, -82.219), I. Agnarson, 31 May 2013, (4, CMNC); **OHIO**: Franklin Co.: Sharon Woods Metro Park (40.12, -82.95), A.J. Penniman, 1973, pitfall (1, ASUIC); Franklin Co.: Columbus (39.97, -83.05), L. Watrous, 13 Sep 1975, berlese (2, CWOB); Licking Co.: Blackhand Gorge (40.056, -82.252), P.W. Kovarik, 3–4 May 1989, berlese leaf litter (1, CMNC); Columbus, Antrim Park (40.078, -83.038), 27 Apr 1989, (1, CMNC). **PENNSYLVANIA**: Allegheny Co.: nr. Sutersville (40.24, -79.80), W. Suter, 16 Jul 1977, litter (2, CWOB). **SOUTH CAROLINA**: Darlington Co.: Great Pee-Dee Heritage Preserve (34.3892, -79.7098), M.S. Caterino, 10 Jan 2017, sifted litter (1, CUAC), DNA extract MSC2484; Greenville Co.: Chestnut Ridge Heritage Preserve (35.1406, -82.279), S.S. Myers, 5 Jun 2015, hardwood litter (3, CUAC), DNA extract SSM096; Greenville Co.: Chestnut Ridge Heritage Preserve (35.1501, -82.282), S.S. Myers, 5 Jun 2015, hardwood litter (2, CUAC), DNA extract SSM092; Greenville Co.: Chestnut Ridge Heritage Preserve (35.1523, -82.2814), S.S. Myers, 5 Jun 2015, hardwood litter (1, CUAC), DNA extract SSM089; Oconee Co.: Coon Branch Natural Area (35.0269, -83.0057), M. & K. Caterino, 28 Feb 2016, sifted hardwood litter (1, CUAC), DNA Extracts SSM465-SSM466; Oconee Co.: Coon Branch Natural Area (35.0272, -83.0069), M. & K. Caterino, 28 Feb 2016, sifted hardwood litter (1, CUAC), DNA Extract SSM467; Oconee Co.: Sumter National Forest, East Fork Trail (34.9838, -83.0979), M.S. Caterino & S.S. Myers, 4 May 2015, sifted litter (1, CUAC), DNA extracts SSM462, SSM463, SSM464; Oconee Co.: Sumter National Forest, East Fork Trail (34.9843, -83.0981), M.S. Caterino & S.S. Myers, 4 May 2015, sifted litter (1, CUAC); Oconee Co.: Sumter National Forest, East Fork Trail (34.9962, -83.1022), S.S. Myers, 29 Jun 2015, sifted litter (1, CUAC), DNA extract SSM317; Oconee Co.: Sumter National Forest, Indian Camp Creek (34.9886, -83.0729), M.S. Caterino & S.S. Myers, 4 May 2015, sifted litter (1, CUAC), DNA extract SSM315; Oconee Co.: Sumter National Forest, Indian Camp Creek (34.9899, -83.0724), M.S. Caterino & S.S. Myers, 4 May 2015, sifted litter (1, CUAC), DNA extract SSM316; Oconee Co.: Sumter National Forest, Indian Camp Creek (34.9903, -83.0723), M.S. Caterino & S.S. Myers, 4 May 2015, sifted litter (2, CUAC), DNA extracts SSM261, SSM262; Oconee Co.: Sumter National Forest, Riley Moore Falls (34.7403, -83.1804), M. Caterino, 3 Mar 2018, sifted litter (1, CUAC), DNA extract MSC2522; Pickens Co.: Eastatoe Heritage Preserve (35.1577, -82.491), S. Myers, 28 Feb 2016, sifted hardwood litter (3, CUAC), DNA Extracts SSM398-SSM400; Pickens Co.: Sassafras Mountain (35.0634, -82.776), S.S. Myers, 10 Jun 2015, sifted hardwood litter (5, CUAC), DNA Extracts SSM080-SSM084; Pickens Co.: Sassafras Mountain (35.0645, -82.7774), S.S. Myers, 10 Jun 2015, sifted hardwood litter (5, CUAC), DNA Extracts SSM075-SSM079. **TENNESSEE**: Blount Co.: Great Smoky Mountains National Park, Sinks Trail (35.67, -83.66), R.T. Allen & R. Chenowith, 13 Oct 1976, leaf litter (1, UAAM); Cocke Co.: Great Smoky Mountains National Park, lower Crosby House (35.7781, -83.2139), P.E. Skelley, 20 Jul 2003, litter at rock wall by stream (1, ASUIC); Marion Co.: Tennessee River Gorge, Pot Point Tr. (35.0864, -85.3922), S. Chatzimanolis, 24 May 2015, sifted litter (2, UTCI); Sevier Co.: Cove Forests S&E Gatlinburg (35.7, -83.5), Hlavac & Lawrence, 10–23 May 1972, litter (1, ASUIC); Sevier Co.: Cove Forests, S.E. Gatlinburg (35.706, -83.5), T. Hlavac; J. Lawrence, 10–23 May 1972, litter (1, CMNC); Sevier Co.: Great Smoky Mountains National Park, Greenbrier Cove (35.71, -83.38), W.B. Suter, 14 Apr 1973, litter at log under rhododendron (1, ASUIC); Sevier Co.: Great Smoky Mountains National Park, Maddron Bald Trail (35.75, -83.27), R.T. Allen & R. Chenowith, 15 Oct 1976, leaf litter (1, UAAM); Sevier Co.: Cove Forests, S & E Gatlinburg (35.64, -83.48), T. Hlavac & J. Lawrence, 10–23 May 1972, litter (6, CWOB); Unicoi Co.: Unaka Mountain (36.148, -82.302), 5 Jul 1953, (1, CMNC); Wilson Co.: Cedars of Lebanon State Park (36.08, -86.315), J.G. Hill, 26 Jul 2010, cedar hardwood litter (2, MEM); Wilson Co.: Cedars of Lebanon State Park (36.08, -86.315), J.M. Campbell, 10 Jun 1997, cedar hardwood litter (1, MEM). **WEST VIRGINIA**: Mercer Co.: Camp Creek State Forest, Mashfork Falls (37.5, -81.14), S. Bird, 24 Feb 1970 (3, CNCI); Putnam Co.: Winfield (38.5, -81.9), L. Torres-Miller, 1 Oct 1996, (2, ASUIC). **WISCONSIN**: Kenosha Co.: Woodworth, Benedicts Prairie (42.55, -88.00), W. Suter, 16 Jun 1976 (1, CWOB); Kenosha Co.: 4.83 km. W. Somers, County Line Forest (42.64, -87.94), W. Suter, 18 Sep 1965, Rhododendron litter (1, CWOB); Racine Co.: 6.44 km. E. Wind Lake (42.83, -88.12), W. Suter, 23 Apr 1966, grassy swamp floor (1, CWOB).

#### Eurhoptus sordidus

Taxon classificationAnimaliaColeopteraCurculionidae

LeConte, 1876

Figures 4 , 5

Acalles sordidus LeConte, 1876: 243; Blatchley and Leng 1916: 501.
Eurhoptus sordidus (part); Blatchley and Leng 1916: 501; Kissinger 1964: 64; O’Brien and Wibmer 1982: 136.

##### Notes about types.

This species was described from a single specimen labelled “Tex”. It has a rectangular label “776” and a red square label with “Type 5268” and a handwritten label “A.sordidus Lec.”. It is not dissected but appears to be a male based on the more coarsely punctate rostral apex. The type was not examined although high resolution images posted on the MCZC Online Type Database (http://140.247.96.247/mcz/Species_record.php?id=5002) were examined. The name *E.sordidus* has been consistently applied to specimens of *Eurhoptus* that were not *E.pyriformis* from Texas east into eastern North America; however, specimens from Texas are distinct from those further east and at least two species under this name have been suggested (Anderson 2002). LeConte’s type came from Gustaf Wilhelm Belfrage who collected insects mainly in Bosque County, Texas, where he lived from 1870–1879 (Geiser 1933). We here apply the name *E.sordidus* to specimens from throughout Texas north into bordering Oklahoma and Arkansas, and east through Louisiana to Mississippi. The herein described new species and the resurrected species closely related to *E.sordidus* occur further to the east and not into Texas although the three are narrowly sympatric in Louisiana and Arkansas. Kissinger (1964) was the first to recognize this species as *Eurhoptus*.

##### Redescription.

Body length (exclusive of head and rostrum) 2.1–2.7 mm, cuticle largely bare, variously covered with short, fine recurved seta-like scales only, although often encrusted and coated with a fine film. Pronotum with lateral margins rounded, margins strongly constricted at about apical one-third such that apical one-third is distinctly tubulate, greatest width at about basal one-third, medially carinate or not. Elytra strongly rounded dorsally and laterally, striae distinct, punctures moderate in size and depth; all elytral intervals evenly elevated, slightly rounded, each with single row of fine recurved seta-like scales. Metaventrite medially impressed behind mesoventral cup. Abdomen with ventrite 1 with pair of obliquely arranged transversely elongate pits which are shallowly continuous medially (but because of encrustation appearing as separate in most specimens), area around pits with dense inwardly directed scales. Legs with femora and tibiae at most fringed with a few erect scales especially along outer margin of tibiae, hind tibiae subequal in width throughout most of length. Aedeagus about as long as one-half length of aedeagal apodemes, in lateral view slightly ventrally curved, in dorsal view with margins subparallel but gradually tapered towards apex at about apical one-third where more strongly tapered, apex not produced medially as narrow extension, broadly rounded at tip. Internal sac with large distinct cruciform apical sclerite complex, with well-defined transverse bar, anterior and posterior bars not well defined.

**Figure 4.**
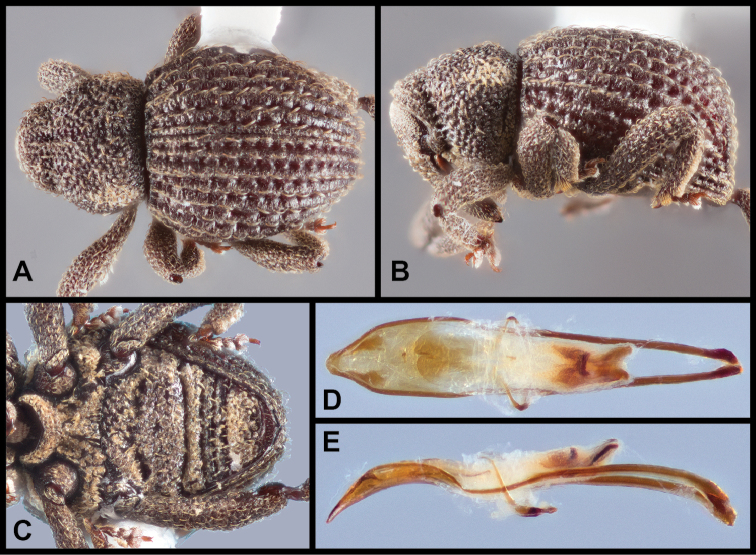
*Eurhoptussordidus*. **A** Dorsal view **B** Lateral view **C** Ventral view showing impressions on ventrites **D** Dorsal view of aedeagus **E** Lateral view of aedeagus.

##### Distribution

**Figure 5.**
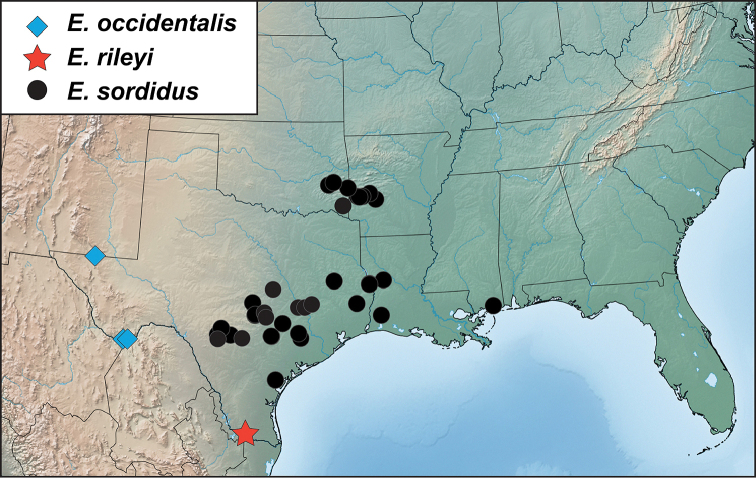
Records of *Eurhoptussordidus*, *E.occidentalis*, and *E.rileyi*.

(Figure 5). This species is distributed from Texas and Oklahoma east into Arkansas and Louisiana with a single outlying specimen from southern Mississippi. Any literature records of this species from the eastern U.S.A. are of *E.curtus* or *E.aenigmaticus*. We have been able to sequence individuals from Arkansas and Texas, and they are very similar and closely related.

##### Specimens examined

(208). **ARKANSAS**: Montgomery Co.: 2.42 km. E. Crystal Recreation Area, slopes at FS 177K (34.5, -93.6), C.E. Carlton & H.W. Robison, 19 Jun 1992, (1, UAAM); Montgomery Co.: Crystal Recreation Area, S. of campground (34.5, -93.6), C.E. Carlton & H.W. Robison, 27 Sep 1991, deciduous litter (1, UAAM); Montgomery Co.: Little Missouri Falls Recreation Area, SW of river (34.4, -93.9), C.E. Carlton & H.W. Robison, 25 Apr 1992, maple beech litter (1, UAAM); Montgomery Co.: (34.29035, -93.35577), C.E. Carlton, (1, CUAC), DNA extract SSM512; Polk Co.: Caney Creek Wilderness Area, 5.63 km. N. Bard Springs (34.4, -94.1), C.E. Carlton & H.W. Robison, 25 Jun 1991, maple beech litter (1, UAAM); Polk Co.: Ouachita National Forest (34.3808, -94.0273), C.E. Carlton & J.S. Park, 16 Oct 2012, leaf litter (2, CUAC; 2, LSAM), DNA extracts SSM491, SSM476, SSM477, SSM492. **LOUISIANA**: Calcasieu Par.: Sam Houston Jones State Park (30.3, -93.267), A. Cline; A. Tishechkin, 11 Feb 2002, leaf litter (2, CMNC); Natchitoches Par.: Kisatchie National Forest, Kisatchie Bayou (31.44, -93.09), A.R. Cline & S. Dash, 20 Sep 2003, sifting (1, CWOB); Natchitoches Par.: Kisatchie National Forest, Kisatchie Bayou (31.44, -93.09), A.R. Cline, 20 Jul 2003, litter/tree holes (3, CWOB); Natchitoches Par.: Kisatchie National Forest. Red Bluff Campground (31.5, -93.14), E.G. Riley; L. Prochaska, 1 Apr 1989, beech-Magnolia-pine litter (1, CMNC). **MISSISSIPPI**: Jackson Co.: Ocean Springs (30.41, -88.828), R.L. Brown, 10 May 1981 (1, MEM). **OKLAHOMA**: Latimer Co.: 11.26 km. S. Wilberton (34.81, -95.31), P.W. Kovarik, 15 Oct 1989, sawdust (1, CMNC); Latimer Co.: (34.9, -95.1), K. Stephan, Mar 1983, (1, ASUIC); Latimer Co.: (34.9, -95.1), K. Stephan, Dec 1981, (1, TAMU); Latimer Co.: (34.9, -95.1), K. Stephan, Feb 1983, (1, TAMU); Latimer Co.: (34.9, -95.1), K. Stephan, Mar 1983, (5, TAMU); Latimer Co.: (34.9, -95.1), K. Stephan, Apr 1983, (5, TAMU); Latimer Co.: (34.9, -95.1), K. Stephan, Nov 1982, (1, ASUIC); Latimer Co.: (34.9, -95.1), K. Stephan, 8 Aug 1990, (1, FSCA); Latimer Co.: (34.9, -95.1), K. Stephan, Dec 1987, (1, FSCA); Latimer Co.: (34.9, -95.1), K. Stephan, Dec 1991, (1, FSCA); Latimer Co.: (34.9, -95.1), K. Stephan, Jul 1992, (1, FSCA); Latimer Co.: (34.9, -95.1), K. Stephan, Jun 1991, (1, FSCA); Latimer Co.: (34.9, -95.1), K. Stephan, May 1990, (1, FSCA); Latimer Co.: (34.9, -95.1), K. Stephan, May 1991, (1, FSCA); Latimer Co.: (34.9, -95.1), K. Stephan, May 1992, (1, FSCA); Latimer Co.: (34.9, -95.1), K. Stephan, Nov 1990, (1, FSCA); Latimer Co.: (34.9, -95.1), K. Stephan, Nov 1991, (1, FSCA); Latimer Co.: (34.9, -95.1), K. Stephan, Sep 1991, (1, FSCA); Latimer Co.: (34.9, -95.1), K. Stephan, Dec 1985, (2, ASUIC); Latimer Co.: (34.9, -95.1), K. Stephan, Dec 1986, (2, ASUIC); Latimer Co.: (34.9, -95.1), K. Stephan, Mar 1986, (2, ASUIC); Latimer Co.: (34.9, -95.1), K. Stephan, 4 Apr 1990, (2, FSCA); Latimer Co.: Buffalo Mountains (34.9, -95.1), K. Stephan, Jun 1991, (2, TAMU); Latimer Co.: Red Oak (34.95, -95.08), K. Stephan, 14 May 1978 (3, CWOB). LeFlore Co.: Rich Mt. (34.7, -94.5), K. Stephan, 24 Feb 1992, (1, FSCA). LeFlore Co.: Ouachita National Forest (34.78, -94.99), K. Stephan, Jul 1994, (2, TAMU). McCurtain Co.: Beaver Bend State Park (34.13, -94.70), W. Suter, 31 Jul 1968, litter (1, CWOB); McCurtain Co.: Beaver Bend State Park (34.13, -94.70), W. Suter, 27 Jul 1968, litter (1, CWOB); McCurtain Co.: Beaver Bend State Park (34.13, -94.70), W. Suter, 27 Jul 1968, litter (1, LSAM). **TEXAS**: Bandera Co.: Hill Country Natural Reserve (29.6, -99.2), C.W. O’Brien & E. Gage, 11 Mar 2001, mixed oak litter (1, ASUIC); Bandera Co.: Lost Maples State Natural Area (29.81958, -99.57066), R.S. Anderson, 28–30 Apr 1988, berlese leaf litter (1, CMNC); Bandera Co.: Lost Maples State Natural Area (29.81958, -99.57066), R.S. Anderson, 28 Apr-2 May 1987, berlese leaf litter (4, CMNC); Bandera Co.: Lost Maples State Natural Area, Maple Trail (29.82, -99.57), C.W. O’Brien & Gages, 10 Mar 2001, litter (1, ASUIC); Bandera Co.: Lost Maples State Natural Area, (29.82, -99.57), C.W. O’Brien, 9 Mar 2001, litter (2, CWOB); Bastrop Co.: Buescher State Park (30.041, -97.162), R.S. Anderson, 3 May 1988, leaf litter berlese (2, CMNC); Bell Co.: Camp Kachina, 6.44 km. W. Temple (31.16, -97.49), E.G. Riley, 5 Mar 1995, leaf litter berlese (1, EGRC); Bexar Co.: San Antonio, Ebony Hill Research Station (29.488, -98.700), E.G. Riley, 9 Feb 1996, berlese forest litter (2, TAMU); Blanco Co.: Pedernales Falls State Park, Nature Trail (30.306, -98.256), R.S. Anderson, 3 May 1988, oak-elm-juniper litter (2, CMNC); Blanco Co.: Pedernales Falls State Park, Twin Falls (30.334, -98.252), R.S. Anderson, 3 May 1988, *Carya*/*Celtis* leaf litter berlese (2, CMNC); Brazos Co.: Ball’s Ferry, 9.66 km. S.W. College Station (30.49, -96.34), E.G. Riley, 26 Nov 2004, cottonwood leaf litter berlese (1, EGRC); Burnett Co.: Inks Lake State Park, Pecan Flat Campground (30.733, -98.364), R.S. Anderson, 21 May 1989, pecan-juniper leaf litter berlese (5, CMNC); Colorado Co.: 1 km. S. Altair on #71 (29.54, -96.45), R.S. Anderson, 1 Feb 1989, live oak/*Ilex* woodland litter berlese (1, CMNC); Colorado Co.: Columbus (29.70665, -96.53534), R.S. Anderson, 4 Nov 1988, riparian ravine hardwood litter (1, CUAC), DNA extract MSC2474; Colorado Co.: Columbus (29.7072, -96.5344), R.S. Anderson, 4 Nov 1988, riparian ravine litter (12, CMNC); Colorado Co.: Columbus (29.7072, -96.5344), R.S. Anderson, 21 Feb 1989, riparian ravine litter (15, CMNC); Colorado Co.: Columbus (29.7072, -96.5344), R.S. Anderson, 22 Mar 1988, riparian ravine litter (2, CMNC); Colorado Co.: Columbus (29.7072, -96.5344), R.S. Anderson, 6 Apr 1988, riparian ravine litter (3, CMNC); Colorado Co.: Columbus (29.7072, -96.5344), R.S. Anderson, 1 Feb 1988, riparian ravine litter (5, CMNC); Colorado Co.: Columbus (29.7072, -96.5344), R.S. Anderson, 17 May 1987, riparian ravine litter (9, CMNC); Gonzales Co.: Palmetto State Park, near East entry (29.59339, -97.58558), R. Anderson, 17–18 Apr 1989, leaf litter berlese (16, CMNC); Grimes Co.: limestone outcrop, 6.44 km. E. Navasota (30.453, -96.026), E.G. Riley, 17 Feb 1996, berlese soil surface litter (5, TAMU); Houston Co.: Big Slough Wilderness Area, FR517 and Four C’s Hiking Trail (31.496, -95.118), R.S. Anderson, 9 May 1988, bottomland hardwood litter (2, CMNC); Houston Co.: Big Slough Wildlife Area, 0.8 km. N. junction FR517 and FR511-3 (31.496, -95.118), R.S. Anderson, 9 May 1988, pine-hardwood litter berlese (1, CMNC); Sabine Co.: 14.5 km. E. Hemphill, Beech Bottom (31.386, -93.706), R. Anderson; E. Riley; E. Morris, 24 Apr 1989, leaf litter berlese (5, CMNC); Sabine Co.: 14.5 km. E. Hemphill, Beech Bottom (31.386, -93.706), E.G. Riley, 16 Mar 1997, litter Berlese beech-Magnolia forest (2, EGRC); Sabine Co.: Hemphill Sabine National Forest, 0.8 km. N. of P. Gomer Rd (31.36489, -93.70636), beech bottom litter (2, CUAC; 2, LSAM), DNA extracts SSM480, SSM481, SSM482, SSM497; San Patricio Co.: Welder Wildlife Refuge (28.118, -97.407), H. & A. Howden; C. Scholtz, 17–25 May 1985, (2, CMNC); San Patricio Co.: Welder Wildlife Refuge (28.118, -97.407), R.S. Anderson, 18–19 Apr 1989, (4, CMNC); San Patricio Co.: Welder Wildlife Refuge, Hackberry Motte (28.118, -97.407), R.S. Anderson, 12–13 Oct 1988, (3, CMNC); San Patricio Co.: Welder Wildlife Refuge, Hackberry Motte (28.118, -97.407), R.S. Anderson, 13–15 Jun 1988, *Celtis* leaf-branch litter (9, CMNC); San Patricio Co.: Welder Wildlife Refuge, 11.26 km. N. Sinton (28.118, -97.406), R.S. Anderson, 18–19 Apr 1989, (1, CUAC), DNA extract MSC2473; San Patricio Co.: Welder Wildlife Refuge, near Hackberry Motte (28.118, -97.407), R.S. Anderson, 1–2 Nov 1988, berlese (1, CMNC); San Patricio Co.: Welder Wildlife Refuge, Big Lake (28.120, -97.372), E.G. Riley, 8 Dec 2007 (2, EGRC); San Patricio Co.: Welder Wildlife Refuge (28.118, -97.407), J.M. Mora, 20 Jul 1989, pitfall trap, live oak woodland (3, TAMU); Travis Co.: Austin, Brackenridge Field Lab (30.284, -97.778), E.G. Riley, 10 Feb 1996, berlese rotten pecan log (1, EGRC); Travis Co.: vic. Long Hollow Creek (30.458, -97.875), 7 May 1994, E.G. Riley, berlese forest litter (2, TAMU); Travis Co.: Shelberg Tract, near Cypress Creek arm of Lake Travis (30.44, -97.87), E.G. Riley, 7 May 1994, berlese forest litter and dung pellets (2, TAMU); Tyler Co.: 4.5 km. W. 3 km. N. Spurger, Big Thicket Natural Preserve, Beech Woods Trail (30.719, -94.227), R.S. Anderson, 8 Mar 1989, beech-*Magnolia* leaf litter berlese (1, CMNC); Uvalde Co.: Garner State Park (29.6, -99.7), C.W. O’Brien, 10 Mar 2001, oak litter (1, ASUIC); Uvalde Co.: Concan, Neal’s Lodge Area (29.494, -99.714), E.G. Riley, 9 Apr 1995, berlese forest litter (6, EGRC); Walker Co.: Sam Houston National Forest, Stubblefield Lake Campground (30.56, -95.64), E.G. Riley, 15 Oct 1995, berlese forest litter (1, EGRC).

#### Eurhoptus curtus

Taxon classificationAnimaliaColeopteraCurculionidae

(Hamilton, 1893), resurrected name

Figures 6 , 7

Acalles curtus Hamilton, 1893: 308; Blatchley and Leng 1916: 501 (as synonym of A.sordidus).
Eurhoptus curtus ; O’Brien and Wibmer 1982: 136 (as synonym of E.sordidus).
Eurhoptus sordidus ; Ciegler 2010: 167.
Eurhoptus sordidus (part); O’Brien and Wibmer 1982: 136; Downie and Arnett 1996: 1581.

##### Notes about types.

This species was described from Pennsylvania from a series of 4 specimens (CMNH) of which we have selected one as lectotype. It is labelled with a handwritten label “Type”, a second rectangular pink blank label, a third rectangular label “Carn. Mus. / Acc. 349”, a fourth red square blank label and our lectotype designation label. It is not dissected but appears to be a male based on sculpture of the rostral apex. This name has long been considered a junior synonym of *E.sordidus* but with the recognition of the latter species as distinct from specimens occurring further east and north, the oldest available name for these eastern forms becomes *E.curtus*.

##### Redescription.

Body length (exclusive of head and rostrum) 1.8–2.8 mm, cuticle largely bare, variously covered with short, fine recurved seta-like scales only, although often encrusted and coated with a fine film. Pronotum with lateral margins rounded, margins constricted at about apical one-third such that apical one-third is tubulate, greatest width at about basal one-third, medially carinate or not. Elytra strongly rounded dorsally and laterally, striae distinct, punctures moderate in size and depth; all elytral intervals evenly elevated, slightly rounded, each with single row of fine recurved seta-like scales. Metaventrite medially impressed behind mesoventral cup. Abdomen with ventrite 1 with pair of obliquely arranged transversely elongate pits which are continuous medially, area around pits with inwardly directed scales. Legs with femora and tibiae at most fringed with a few erect scales especially along outer margin of tibiae, hind tibiae subequal in width throughout most of length. Aedeagus about as long as one-half length of aedeagal apodemes or slightly shorter, in lateral view slightly ventrally curved, in dorsal view with margins very gradually tapered towards apex at about apical one-quarter to one-fifth where abruptly constricted, apex produced medially as broad lobe-like extension, broadly rounded at tip. Internal sac with large distinct cruciform apical sclerite complex, with well-defined posterior bars, anterior and transverse bars not well defined.

**Figure 6.**
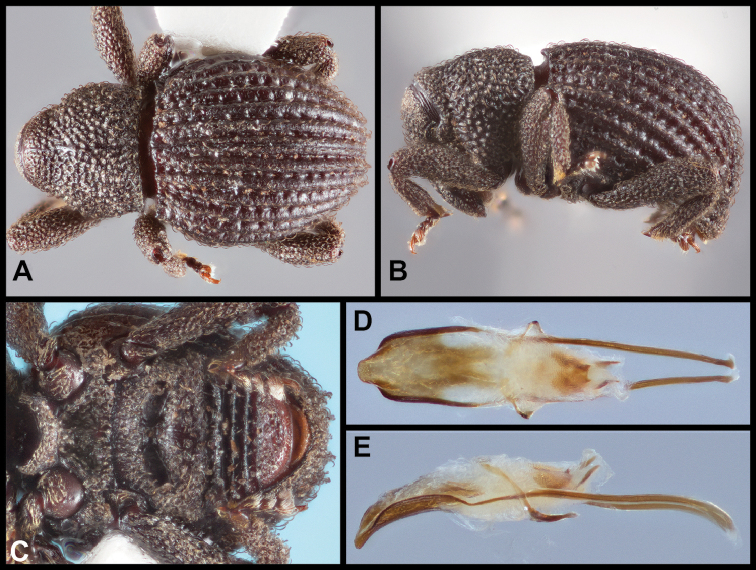
*Eurhoptuscurtus*. **A** Dorsal view **B** Lateral view **C** Ventral view showing impressions on ventrites **D** Dorsal view of aedeagus **E** Lateral view of aedeagus.

##### Distribution

**Figure 7.**
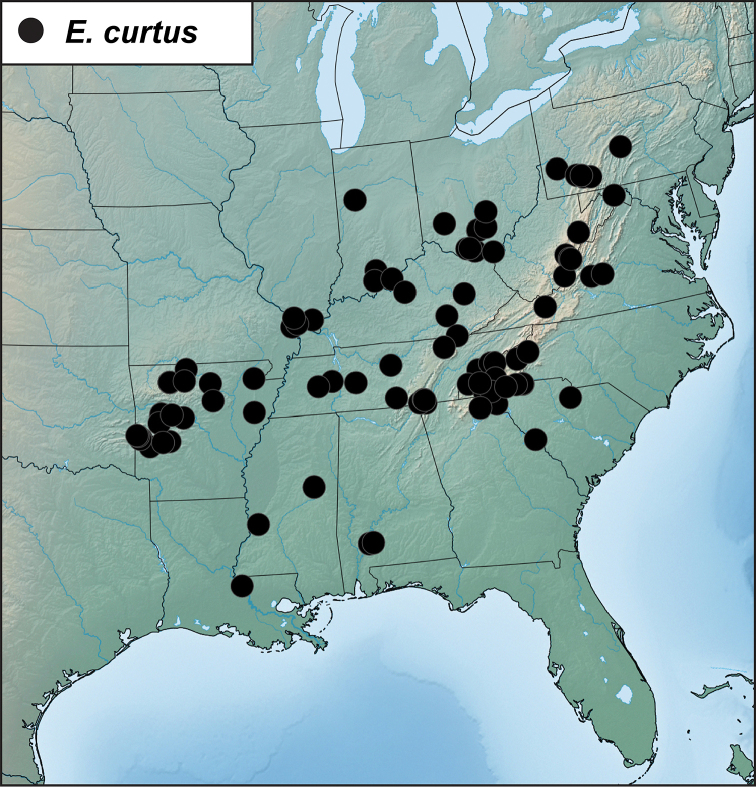
Records of *Eurhoptuscurtus*.

(Figure 7). This species is widely distributed in the eastern U.S.A. from Illinois and Pennsylvania in the north, south nearly to the Gulf Coasts of Louisiana and Mississippi. Across this range it exhibits a high degree of intraspecific genetic variation, with some surprisingly well supported subclades, especially in a group of populations from central South Carolina (Greenville and McCormick Counties). However, most of the range of the species is sparsely represented in the sequence data set, and no further subdivision appears warranted.

##### Specimens examined

(219). **ALABAMA**: Monroe Co.: 1.6 km. S. Claiborne Dam (31.5917, -87.5392), C.E. Carlton, May 31 1995, beech/magnolia riparian berlese (1, ASUIC); Monroe Co.: Big Flat Creek (31.6083, -87.4147), C.E. Carlton, May 27 1995, riparian litter (1, ASUIC); Monroe Co.: Haines Island Park (35.7231, -87.4694), K. Schnepp, Jun 12 2016, leaf litter (1, KSC), DNA extract MSC2515; Monroe Co.: Haines Island Park (35.7231, -87.4694), C.E. Carlton, May 31 1995, beech/magnolia berlese (1, ASUIC). **ARKANSAS**: Cleburne Co.: Greers Ferry Dam, Mossy Bluff Trail (35.52, -91.99), R. Chenowith, 30 Jun 1976, litter (1, CWOB); Conway Co.: Petit Jean State Park (35.118, -92.942), J.A. MacGown; J.G. Hill, Aug 5 2008, litter hardwood forest (6, MEM); Cross Co.: Village Creek State Park (35.168, -90.721), R.S. Anderson, Aug 14 1988, hardwood berlese (1, CMNC); Garland Co.: 4.83 km. W. Crystal Springs (34.521, -93.389), J. Pakaluk, Jun 1 1984, hardwood berlese (1, CMNC); Greene Co.: Crowleys Ridge State Park (36.04, -90.67), R. Chenowith, 8 Jun 1976, litter (1, CWOB); Izard Co.: 20.9 km. S. Melbourne (35.97, -92.05), R. Chenowith, 30 Aug 1977, hardwood litter (2, CWOB); Logan Co.: Cove Lake Recreation Area (35.228, -93.627), C.E. Carlton, 16 Jun 1990, berlese (2, CMNC; 2 LSAM); Logan Co.: Mount Magazine Road, 1.6 km. N. Greenfield intersection (35.167, -93.645), C. Carlton, May 21 1987, hardwood berlese (3, CMNC); Logan Co.: Mount Magazine State Park (35.1686, -93.6428), A.P.G. Dowling, Jun 11 2013, oak/hickory litter (1, UAAM); Logan Co.: Mount Magazine State Park (35.1686, -93.6428), J.R. Fisher & M.J. Skvarla, May 25 2013, oak/hickory litter (1, UAAM); Logan Co.: Mount Magazine State Park (35.1919, -93.6447), A.P.G. Dowling, Sep 22 2014, paw-paw/sassafras litter (1, UAAM), DNA extract MSC2527; Logan Co.: Mount Magazine, Signal Hill Trail (35.17, -93.64), E.G. Riley, 13 May 1995 (1, EGRC); Marion Co.: 7 mi E County line on Hwy 14 (36.36, -92.8), R. Chenowith, Sep 27 1977, mixed hardwood litter (1, ASUIC); Montgomery Co.: 2.41 km. E. Crystal Recreation Area, slopes at FS 177K (34.5, -93.6), C.E. Carlton & H.W. Robison, Aug 17 1991, deciduous litter (1, UAAM); Newton Co.: Buffalo National River (36.0369, -93.34), M.J. Skvarla, Aug 16 2013, litter (1, UAAM); Newton Co.: Buffalo National River (36.0369, -93.34), M.J. Skvarla, Jul 31 2013, litter along log (1, CUAC), DNA extract MSC2526; Newton Co.: Buffalo National River (36.0369, -93.34), M.J. Skvarla, May 29 2013, oak/hickory litter (1, UAAM); Newton Co.: Buffalo National River (36.0372, -93.3417), M.J. Skvarla, Sep 25 2013, litter next to large branch (1, UAAM); Newton Co.: Buffalo National River (36.0375, -93.34), M.J. Skvarla, May 29 2013, pitfall (1, UAAM); Newton Co.: Buffalo National River (36.0386, -93.3411), M.J. Skvarla, Apr 13 2013, pitfall in grassy area (1, UAAM); Newton Co.: Buffalo National River (36.0386, -93.34), M.J. Skvarla, Aug 28 2013, pitfall (1, UAAM); Newton Co.: Buffalo National River (36.0386, -93.34), M.J. Skvarla, May 15 2013, pitfall (1, UAAM); Newton Co.: Buffalo National River (36.0386, -93.3411), M.J. Skvarla, May 29 2013, oak/hickory litter (1, UAAM); Newton Co.: Buffalo National River (36.0386, -93.3417), M.J. Skvarla, Sep 25 2013, litter in short shrubs (1, UAAM); Newton Co.: Buffalo National River (36.0389, -93.3401), M.J. Skvarla, Apr 30 2013, litter in blueberry (1, UAAM); Newton Co.: Buffalo National River (36.0389, -93.3408), M.J. Skvarla, Aug 16 2013, litter (1, UAAM); Newton Co.: Buffalo National River (36.0389, -93.34), M.J. Skvarla, Aug 28 2013, pitfall (1, UAAM); Newton Co.: Buffalo National River (36.0389, -93.3408), M.J. Skvarla, Jul 17 2013, litter under low bush (1, UAAM); Newton Co.: Buffalo National River (36.0389, -93.3403), M.J. Skvarla, Sep 25 2013, thick litter in flats (1, UAAM); Newton Co.: Buffalo National River (36.0392, -93.3411), M.J. Skvarla, Jul 17 2013, litter in low bushes (1, UAAM); Newton Co.: Buffalo National River (36.0394, -93.3401), M.J. Skvarla, Aug 16 2013, litter (1, UAAM); Perry Co.: Cedar Creek Gorge, ca. 19.3 km. W. Oppelo (35.12, -92.94), E.G. Riley, 14–16.Oct 1999, berlese forest litter (5, EGRC); Polk Co.: Bard Springs Recreation Area (34.4, -94), C.E. Carlton & H.W. Robison, Apr 24 1992, deciduous litter (1, UAAM); Polk Co.: Bard Springs Recreation Area (34.4, -94), C.E. Carlton & H.W. Robison, Jun 6 1992, deciduous litter (1, UAAM); Polk Co.: Rich Mt., Eagleton Overlook (34.6, -94.3), C.E. Carlton & H.W. Robison, Nov 29 1991, deciduous litter (1, UAAM); Polk Co.: Rich Mt., intersec 272-88 (34.68, -94.36), C.E. Carlton & H.W. Robison, Apr 3 1992, deciduous litter (1, UAAM); Polk Co.: Rich Mt., intersec 272-88 (34.68, -94.36), C.E. Carlton & H.W. Robison, Nov 16 1991, deciduous litter (1, UAAM); Polk Co.: Rich Mt., intersec 272-88 (34.68, -94.36), C.E. Carlton & H.W. Robison, Oct 18 1991, deciduous litter (1, UAAM); Polk Co.: Rich Mt., Ouachita Trail (34.7, -94.4), C.E. Carlton & H.W. Robison, May 3 1992, deciduous litter (1, UAAM); Polk Co.: Rocky, Talimena Drive (34.619, -94.276), D. Beyers; M. Jenks, Apr 14 1984, hardwood berlese (2, CMNC); Pope Co.: Mt. Nebo State Park, entrance area (35.2, -93.3), C.E. Carlton & R.A.B. Leschen, Apr 16 1989, hardwood berlese (1, UAAM); Scott Co.: Dry Creek Wilderness Area (35, -93.7), C.E. Carlton & H.W. Robison, Jan 19 1992, deciduous litter (1, UAAM); Searcy Co.: Hwy 853.9 mi E County line (36.06, -92.87), R. Chenowith, Mar 25 2977, (1, ASUIC); Yell Co.: 6.44 km. S. Blue Mt. Lake (35, -93.7), R. Chenowith, Aug 16 1991, hardwood berlese (1, ASUIC); Yell Co.: Dry Creek Wilderness Area (35, -93.7), C.E. Carlton & H.W. Robison, Mar 6 1992, deciduous litter (1, UAAM). **GEORGIA**: White Co.: (34.7, -83.7), R. Morris II, Jun 19 1984, (1, FSCA). **ILLINOIS**: Alexander Co.: Shawnee National Forest, nr. McClure (37.2894, -89.3427), M.L. Ferro, May 17 2016, sifted forest litter (2, CUAC), DNA extract SSM504; Pope Co.: Shawnee National Forest. Dixon Springs Agricultural Centre (37.434, -88.667), I. Askevold, Jun 4 1983, (1, CMNC); Union Co.: Cobden (37.52, -89.26), H.S. Dybas, 23 Aug 1960, litter (1, CWOB); Union Co.: Dongola (37.36, -89.17), D. Dillo, 15 Aug 1968, litter (1, CWOB); Union Co.: Pine Hills Field Station (37.55, -89.43), J.M. Campbell, 15–22 May 1967 (10, CNCI). **INDIANA**: Crawford Co.: Hemlock Cliffs Trail (38.2785, -86.5393), R.S. Anderson, Sep 30 2017, mixed hardwood litter (1, CUAC), DNA extract MSC2479-2480, 2487-2488; Floyd Co.: rest stop nr. Georgetown (38.2775, -85.9626), M.L. Ferro, May 13 2016, sifted forest litter (2, CUAC), DNA extract SSM511; Orange Co.: Pioneer Mothers Memorial Forest (38.5356, -86.4568), R.S. Anderson, Sep 30 2017, mixed hardwood litter (1, CUAC), DNA extract MSC2477-2478, 2485-2486; Tippecanoe Co.: (40.4, -86.9), K. Schnepp, Mar 22 2009, leaf litter (1, KSC), MSC2514. **KENTUCKY**: Bull Co.: Bernheim Forest, Clermont (37.9, -85.6), R.M. Reeves, Dec 7 1988, box elder leaf litter (1, ASUIC); Christian Co.: 8.05 km. W. Hopkinsville (36.86. -87.41), J.M. Campbell, 22 Sep 1967 (5, CNCI); Edmonson Co.: Mammoth Cave National Park, Cabin Woods (37.20, -86.10), W. Suter, 24 Mar 1973, litter (1, CWOB); Laurel Co.: 24.14 km. W. London (37.161, -84.35), S. Marshall, Jun 4–30 1984, (2, CMNC); Lee Co.: Hwy. 11 2 km S. Zoe (37.6607, -83.6966), M.L. Ferro, May 11 2016, sifted forest litter (1, CUAC), DNA extract SSM508; Whitley Co.: I-75 Welcome Center (36.6144, -84.1047), M.L. Ferro, May 11 2016, forest litter (1, CUAC), DNA extract SSM509. **LOUISIANA**: West Feliciana Parish: St. Francisville (30.76, -91.39), S. McGlynn, Feb 9 1999, beech/magnolia litter near lake (1, ASUIC). **MISSISSIPPI**: Warren Co.: Vicksburg (32.3, -90.8), C.W. O’Brien & P.W. Kovarik, Apr 24 1996, beech/magnolia berlese (1, ASUIC); Winston Co.: Legion State Park (33.155, -89.045), J.A. MacGown; J.G. Hill, Aug 4 2006, berlese litter (2, MEM). **NORTH CAROLINA**: Buncombe Co.: Big Butt Trail (35.80174, -82.3401), S.S. Myers, Jul 26 2015, sifted litter (1, CUAC), DNA extract SSM326; Graham Co.: Joyce Kilmer Memorial Forest (35.3441, -83.966), M.S. Caterino & S.S. Myers, Jun 24 2015, sifted litter (1, CUAC), DNA extract SSM018; Jackson Co.: Balsam Mountain Preserve (35.3653, -83.1062), S.S. Myers, Jun 17 2015, sifted litter (1, CUAC), DNA extract SSM222; Jackson Co.: Balsam Mountain Preserve (35.3681, -83.1036), S.S. Myers, Jun 17 2015, sifted litter (1, CUAC), DNA extract SSM388; Jackson Co.: Balsam Mountain Preserve (35.3691, 83.1191), S.S. Myers, Jun 15 2015, sifted litter (1, CUAC), DNA extract SSM547; Jackson Co.: Balsam Mountain Preserve (35.3699, -83.1212), S.S. Myers, Jun 15 2015, sifted litter (1, CUAC), DNA extract SSM546; Jackson Co.: Balsam Mountain Preserve (35.37, -83.1216), S.S. Myers, Jun 15 2015, sifted litter (1, CUAC), DNA extract SSM545; Jackson Co.: Balsam Mountain Preserve (35.372, -83.0998), S.S. Myers, Jun 17 2015, sifted litter (3, CUAC), DNA extract SSM387; Jackson Co.: Balsam Mountain Preserve (35.3916, -83.1091), S.S. Myers, Jun 17 2015, sifted litter (1, CUAC), DNA extract SSM355; Jackson Co.: Balsam Mountain Preserve (35.398, -83.1088), S.S. Myers, Jun 17 2015, sifted litter (2, CUAC), DNA extract SSM225; Jackson Co.: Balsam Mountain Preserve (35.4025, -83.1049), S.S. Myers, Jun 17 2015, sifted litter (2, CUAC), DNA extract SSM228; Macon Co.: Coweeta Hydrological Station, Shope Fork Rd. (35.1, -83.5), D.S. Chandler, May 30 1983, oak and ash litter (1, ASUIC); Macon Co.: Jones Gap (35.0785, -83.2923), S.S. Myers, Jul 22 2015, sifted litter (1, CUAC), DNA extract SSM178; McDowell Co.: Blue Ridge Parkway, mile 320 (35.94, -81.96), Q. Wheeler, Aug 4 1981, leaf litter (1, ASUIC); Swain Co.: Miller cove, Appalachian Trail (35.3367, -83.6021), S.S. Myers, Jul 20 2015, sifted litter (2, CUAC), DNA extract SSM351; Transylvania Co.: 1.6 km. S. Rosman (35.14, -82.82), J. Cornell, 26 Apr 1988, litter (1, CWOB); Co.: Great Smoky Mountains National Park, Chestnut Branch Trail (35.76, -83.123), A. Tishechkin, Aug 1 2001, forest leaf litter (3, CMNC). **OHIO**: Adams Co.: 1 mi W Lynch (38.8, -83.4), L.E. Watrous, Sep 3 1978, litter rotten log (3, ASUIC); Hocking Co.: 11.26 km. S. Lancaster (39.67, -82.60), L.E. Watrous, 4 Jun 1977, litter around swamp (1, CWOB); Lawrence Co.: Wayne National Forest, Sharps Creek, Bluegrass Trail (38.62, -82.56), Jun 1 1995, pitfall (1, ASUIC; 1, CWOB); Preble Co.: Hueston Woods State Park (35.814, -88.209), P. Kovarik, May 19 1990, (1, CMNC); Ross Co.: Scioto State Park (39.23, -82.95), L.E. Watrous, Sep 4 1978, leaf litter (1, ASUIC); Ross Co.: Scioto Trail State Park (39.23, -82.956), P. Kovarik, Apr 17 1989, (1, CMNC); Scioto Co.: Shawnee State Forest, 12.87 km. W. Portsmouth (38.71, -83.15), P. Kovarik, Apr 22 1989, (9, CMNC); Vinton Co.: 8.05 km. N. Ratcliffburg (39.28, -82.68), L.E. Watrous, 15 May 1977, litter along stream (2, CWOB); Warren Co.: Caesar Creek State Park (39.519, -84.025), P. Kovarik, May 19 1990, (4, CMNC). **PENNSYLVANIA**: Huntingdon Co.: Rothrock State Forest, Seeger Natural Area (40.69, -77.76), D.S. Chandler, May 30 1985, mixed leaf litter (1, ASUIC); Somerset Co.: 1.6 km. S. Kantner (40.09, -78.94), L.E. Watrous, 10 Jun 1976, litter (1, CWOB); Westmoreland Co.: Chestnut Ridge (40.2, -79.4), W. Suter, Sep 29 1978, litter at log (1, ASUIC); Westmoreland Co.: Forbes State Forest, Laurel summit (40.12, -79.18), D.S. Chandler, Jul 10 1984, oak litter (1, ASUIC); Westmoreland Co.: Linn Run State Park, Adams Falls (40.17, -79.23), D.S. Chandler, Jul 11 1984, leaf litter at stream edge (1, ASUIC); Westmoreland Co.: Linn Run State Park, Linn Run (40.17, -79.23), D.S. Chandler, Jul 12 1984, leaf litter (1, ASUIC); Allegheny (40.45, -80.01), J. Hamilton, Apr , under stones near beech trees (3, CMNH). **SOUTH CAROLINA**: Chester Co.: nr. Great Falls on US 21 at Rockey Creek Bridge (34.60, -80.89), J. & S. Cornell, 28 Mar 2009, old stream debris (2, CWOB); Greenville Co.: Chestnut Ridge Heritage Preserve (35.1406, -82.279), S.S. Myers, Jun 5 2015, hardwood litter (4, CUAC), DNA extract SSM540; Greenville Co.: Chestnut Ridge Heritage Preserve (35.1518, -82.2839), S.S. Myers, Jun 5 2015, hardwood litter (3, CUAC), DNA extract SSM87; Greenville Co.: Chestnut Ridge Heritage Preserve (35.1523, -82.2814), S.S. Myers, Jun 5 2015, hardwood litter (5, CUAC), DNA extract SSM90; McCormick Co.: Stevens Creek Heritage Preserve (33.6893, -82.1603), M.S. Caterino, S.S. Myers & L.Vasquez-Velez, Feb 14 2016, sifted litter (4, CUAC), DNA extract SSM549; Oconee Co.: Sumter National Forest, Riley Moore Falls (34.7403, -83.1804), M.S. Caterino & S.S. Myers, Mar 3 2018, sifted litter (1, CUAC), DNA extract MSC2523; Pickens Co.: Eastatoe Heritage Preserve (35.1577, -82.491), S.S. Myers, Jun 30 2015, sifted litter (1, CUAC), DNA extract SSM397. **TENNESSEE**: Cocke Co.: 6 mi SE Cosby (35.8, -83.2), D.S. Chandler, May 31 1983, forest litter (1, ASUIC); Cocke Co.: Snowbird Mt. (35.79, -83.05), K. Schnepp, Oct 12 2014, sifted litter (2, KSC), DNA extracts MSC2512, MSC2513; Franklin Co.: Tims Ford State Park (35.222, -86.253), J.A. MacGown, Jun 17 2010, soil/litter hardwood forest (1, MEM); Hamilton Co.: Chattanooga Nature Centre (35.0052, -85.3648), S. Chatzimanolis et al. , 30 Apr-1 May 2010, leaf litter (2, CMNC); Hamilton Co.: Chattanooga Nature Centre (35.0052, -85.3648), S. Chatzimanolis et al. , May 21 2011, Magnolia leaf litter (1, CMNC); Hamilton Co.: Tennessee River Gorge (35.08643, -85.39215), S. Chatzimanolis, Oct 20 2012, leaf litter (1, CMNC); Henderson Co.: I40, 3.2 km. E. Natchez Trace State Park (35.814, -88.209), R.S. Anderson, Jun 24 1988, hardwood litter (1, CMNC); Madison Co.: Route 152, 1.6 km. W. I40 (35.71, -88.659), P. Kovarik, Mar 6 1988, (2, CMNC); Marion Co.: near Nicklejack Lake (35.029, -85.549), S. Chatzimanolis et al. , Nov 25 2009, leaf litter (2, CMNC); Marion Co.: Tennessee River Gorge (35.1046, -85.4122), S. Chatzimanolis, Jun 15 2015, sifted litter (2, CUAC); Marion Co.: Tennessee River Gorge, Pot Point Tr. (35.0864, -85.3922), S. Chatzimanolis, May 24 2015, sifted litter (3, CUAC), DNA extract MSC2521; Scott Co.: 1.6 km. S. New River (36.37, -84.57), H.S. Dybas, 20 Dec 1969, litter (1, CWOB); Sevier Co.: Great Smoky Mountains National Park (35.679, -83.6), J.A. MacGown; J.G. Hill, Jun 9 2009, litter old growth hardwood forest (1, MEM); Wilson Co.: Cedars of Lebanon State Park (36.08, -86.315), J.G. Hill, Jun 4 2010, cedar hardwood litter (3, MEM). **VIRGINIA**: Boutetourt Co.: Cave Mountain Lake Campground, 404 m (37.566, -79.54), I. Agnarson, May 28 2013, (2, CMNC). **WEST VIRGINIA**: Greenbrier Co.: Greenbrier State Park (37.7, -80.4), W. Suter, Jul 8 1982, litter at birch log (1, ASUIC); Morgan Co.: Cacapon State Park (39.5, -78.3), D.S. Chandler, May 31 1983, litter near stream (1, ASUIC); Pendleton Co.: George Washington National Forest, High Knob Trail (37.57, -79.17), K. Schnepp, May 9 2017, under rock (2, KSC), DNA extracts MSC2510, MSC2511; Pendleton Co.: George Washington National Forest, High Knob Trail (37.57, -79.17), K. Schnepp, May 9 2017, fermenting sugar pitfall (1, KSC); Pendleton Co.: George Washington National Forest, High Knob Trail (37.57, -79.17), K. Schnepp, May 10 2017, leaf litter (1, KSC); Pocahontas Co.: Watogas State Park, 3.2 km. NE Seebert (38.1, -80.1), W. Suter, Jul 8 1982, under rhododendron (2, ASUIC); Pochahontas Co.: Highway 150, Cranberry Glades Overlook (38.23, -80.234), P. Kovarik, Jun 8 1990, (2, CMNC); Pochahontas Co.: Watogas State Park, 3.2 km. N.E. Seebert (38.121, -80.138), W. Suter, Jul 8 1982, (1, CMNC); Pochahontas Co.: Hills Creek Falls (38.17, -80.34), W. Shear, 19 Jun 1971 (1, CNCI); Randolph Co.: Monongahela National Forest, Laurel Fork Campground, 855 m (38.74, -79.693), I. Agnarson, May 27 2013, (1, CMNC); Summers Co.: 19.3 km. N. Athens (37.59, -80.97), W.A. Shear, 16 Jun 1971 (1, CNCI); Wythe Co.: Stoney Fork Campground, 713 m (37.009, -81.182), I. Agnarson, May 29 2013, (1, CMNC).

#### Eurhoptus imbricatus

Taxon classificationAnimaliaColeopteraCurculionidae

Anderson & Caterino sp. n.

http://zoobank.org/3AFF4D3A-E68D-4DFF-97F2-732E2E0BFAF9

Figures 8 , 9

##### Description.

Body length (exclusive of head and rostrum) 2.3–3.0 mm, cuticle almost fully covered with broad, flat scales and scattered short, fine recurved (pronotum) to appressed (elytra) seta-like scales. Pronotum with lateral margins rounded, margins constricted at about apical one-third such that apical one-third is tubulate, greatest width at about basal one-third, not medially carinate; pale scales almost entirely covering pronotum to limited to midline and pair of lateral lines (giving some specimens a trivittate appearance). Elytra strongly rounded dorsally and laterally, striae distinct, of small punctures; all elytral intervals flat, each with single row of fine almost appressed seta-like scales; broad flat scales largely dark brown, approximate, scales pale and imbricate in an irregular oblique band across elytra in apical one-half, at bases of interval 3, at humeri and at elytral apex. Metaventrite medially impressed behind mesoventral cup. Abdomen with ventrite 1 with large median deep shining pit, base of pit with numerous erect, broad pale scales, scales somewhat condensed along midline in anterior portion of pit (giving a divided appearance); area around pit with dense, fine golden inwardly directed scales encircling depression. Legs with femora and tibiae fringed with dense erect scales along outer and inner margins, hind tibiae distinctly wider at about basal one-third. Aedeagus about as long as slightly less than one-half length of aedeagal apodemes, in lateral view slightly ventrally curved, in dorsal view with margins subparallel but gradually rounded towards apex at about apical one-quarter, apex produced medially as slight extension, broadly rounded at tip. Internal sac with apical sclerite complex with well-defined pair of hook-like sclerites.

**Figure 8.**
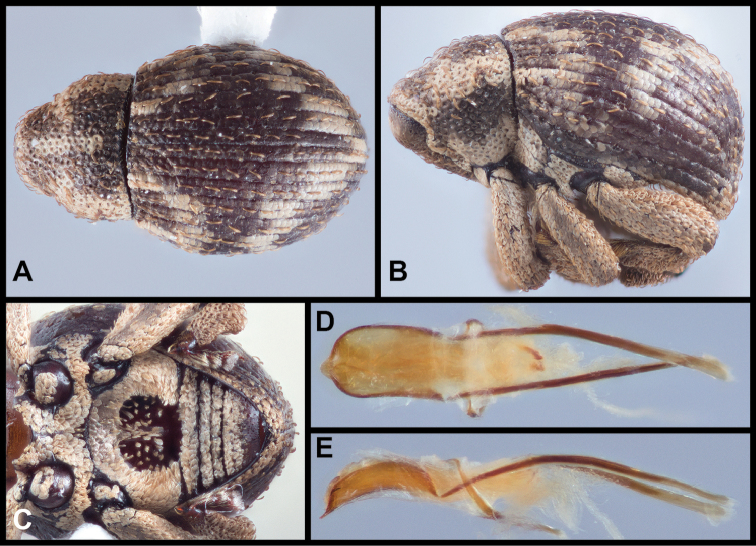
*Eurhoptusimbricatus*. **A** Dorsal view **B** Lateral view **C** Ventral view showing impressions on ventrites **D** Dorsal view of aedeagus **E** Lateral view of aedeagus.

##### Distribution

**Figure 9.**
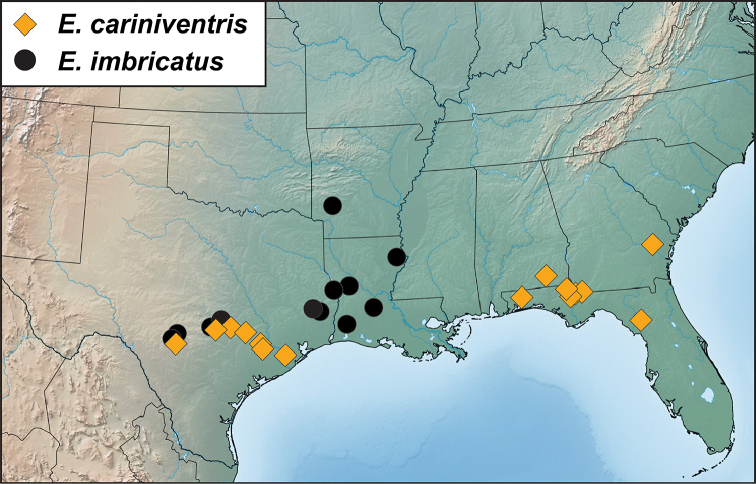
Records of *Eurhoptusimbricatus* and *E.cariniventris*.

(Figure 9). This species is distributed in the Edwards Plateau region of central Texas, east into Louisiana and southern Arkansas.

##### Etymology.

We name this species “imbricatus” (L., an adjective or participle in the nominative singular, tiled, having adjacent edges overlapping) after the overlapping or imbricate scales of the oblique band on the elytra.

##### Variation.

There is some variation in the extent of the distribution of pale scales on the pronotum.

##### Specimens examined.

Holotype male (CMNC), labelled “USA: TX: Bandera Co. / 29.81958°N, 99.57066°W / Lost Maples St. Nat. Area / IV.28–30.1988, leaflit. / Berlese, R.S. Anderson”, dissected. Extracted MSC2469 (unsuccessful). Paratypes (125): **TEXAS**: Bandera Co.: Lost Maples State Natural Area (29.81958, -99.57066), R.S. Anderson, 28–30 Apr 1988, berlese leaf litter (11, CMNC; 2, CNCI; 1, CWOB; 4, TMMC); Bandera Co.: Lost Maples State Natural Area (29.81958, -99.57066), R.S. Anderson, 28 Apr-2 May 1987, leaf / log litter (4, BMNH; 13, CMNC; 1, CWOB; 4 USNM); Bandera Co.: Lost Maples State Natural Area (29.82, -99.57), P.W. Kovarik, 21 Apr 1986, leaf litter (1, FSCA; 2, TAMU); Bandera Co.: Lost Maples State Natural Area (29.82, -99.57), P.W. Kovarik, 26 Apr 1986, leaf litter (2, TAMU); Bandera Co.: Lost Maples State Natural Area (29.82, -99.57), R.S. Anderson, 28–30 Apr 1988, leaf litter (1, CUAC); Bandera Co.: Lost Maples State Natural Area (29.82, -99.57), E.G. Riley, 27 Mar 1999, forest litter (1, EGRC); Bandera Co.: Lost Maples State Natural Area, Maple Trail (29.82, -99.57), C.W. O’Brien, 9 Mar 2001, leaf litter (5, CWOB); Bandera Co.: Lost Maples State Natural Area, Maple Trail (29.82, -99.57), C.W. O’Brien, 10 Mar 2001, leaf litter (8, CWOB); Bandera Co.: Lost Maples State Natural Area (29.82, -99.57), C.E. Carlton, 19 May 2005, forest litter (2, LSAM); Hays Co.: 9.66 km. N.W. Dripping Springs (30.226, -98.184), E.G. Riley, 24 Feb-30 Mar 2006, pitfall, *Juniperus*, unmanaged plot (2, EGRC); Hays Co.: 9.66 km. N.W. Dripping Springs (30.226, -98.184), E.G. Riley, 28 Apr-2 Jun 2006, FIT *Juniperus*, unmanaged plot (1, EGRC); Hays Co.: 9.66 km. N.W. Dripping Springs (30.226, -98.184), E.G. Riley, 31 arr-26 Apr 2006, FIT *Juniperus*, unmanaged plot (1, EGRC); Kerr Co.: 10.46 km. S.W. Hunt (29.99, -99.387), E.G. Riley, 28 Apr-2 Jun 2006, pitfall, upland deciduous forest (1, EGRC); Sabine Co.: 14.48 km. E. Hemphill ‘Beech Bottom’ (31.38, -93.71), R.S. Anderson, 11 May 1988, beech/magnolia berlese (1, CUAC); Sabine Co.: 14.48 km. E. Hemphill ‘Beech Bottom’ (31.38, -93.71), R.S. Anderson & E. Morris, 8 Mar 1989, beech/magnolia berlese (1, CUAC); Sabine Co.: 14.48 km. E. Hemphill ‘Beech Bottom’ (31.38, -93.71), E. Riley & E. Morris, 24 Apr 1989, beech/magnolia berlese (1, CUAC); Sabine Co.: 14.48 km. E. Hemphill, Beech Bottom (31.386, -93.706), R.S. Anderson, 11 May 1988, litter, beech-magnolia forest (10, CMNC; 1, CWOB); Sabine Co.: 14.48 km. E. Hemphill, Beech Bottom (31.386, -93.706), R. Anderson; E. Morris, 8 Mar 1989, litter, beech-magnolia forest (9, CMNC; 1, CWOB); Sabine Co.: 14.48 km. E. Hemphill, Beech Bottom (31.386, -93.706), R. Anderson; E. Riley; E. Morris, 24 Apr 1989, litter, beech-magnolia forest (6, CMNC); Sabine Co.: 14.48 km. E. Hemphill, Beech Bottom (31.386, -93.706), E. Morris; R. Anderson, 18–26 Apr 1989, FIT, beech-magnolia forest (1, CMNC); Sabine Co.: 14.48 km. E. Hemphill, Beech Bottom (31.386, -93.706), E. Morris; R. Anderson, 9–16 Apr 1989, FIT, beech-magnolia forest (3, CMNC); Tyler Co.: 4.5 km. W., 3.05 km. N. Spurger, Big Thicket Natural Preserve, Beech Woods Trail (30.72, -94.227), R.S. Anderson, 12 May 1988, beech/magnolia leaf litter (6, CMNC); Travis Co.: vic. Long Hollow Creek (30.458, -97.875), 17 Mar 1994, E.G. Riley, berlese forest litter (2, TAMU); Travis Co.: vic. Long Hollow Creek (30.458, -97.875), 7 May 1994, E.G. Riley, berlese forest litter (3, TAMU); Tyler Co.: 4.5 km. W., 3.05 km. N. Spurger, Big Thicket Natural Preserve, Beech Woods Trail (30.72, -94.227), R.S. Anderson, 24 Apr 1988, beech/magnolia leaf litter (3, CMNC); Tyler Co.: Big Thicket National Preserve, 4.5 km. W, 3.05 km. N. Spurger, Beech Woods Trail (30.72, -94.23), R.S. Anderson, 24 Apr 1988, beech/magnolia berlese (1, CUAC); Tyler Co.: Highway 190 at FM 256 (30.76, -94.50), R.H. Turnbow Jr., 8 Feb 2003, berlese leaf litter (1, CWOB). **ARKANSAS**: Pike Co.: Crater of Diamonds State Park (34.04, -93.67), R.S. Anderson, 13 Aug 1988, hardwood berlese (1, CMNC). **LOUISIANA**: Calcasieu Par.: Sam Houston Jones State Park (30.3, -93.27), A. Cline; A. Tishechkin, 11 Feb 2002, leaf litter (2, CMNC); Evangeline Par.: Chicot State Park (30.8, -92.28), A. Tishechkin, 19 Feb 2004, berlese (1, CMNC); Evangeline Par.: Chicot State Park (30.794, -92.278), A.K. Tishechkin, 19 Feb 2004, berlese (1, CUAC); Madison Par.: Tallulah, Tensas National Wildlife Refuge (32.34, -91.34), S.T. Dash, 24 Mar 2004, leaf litter (1, CMNC); Natchitoches Par.: Kisatchie National Forest, Red Bluff Camp (31.5, -93.14), E. Riley; L. Prochaska, 1 Apr 1989, berlese beech/magnolia/pine litter (1, CMNC); Pointe Coupee Par.: Sam Houston Jones State Park (30.297, -93.259), 19 Feb 2003, A.R. Cline (1, CWOB).

#### Eurhoptus rileyi

Taxon classificationAnimaliaColeopteraCurculionidae

Anderson & Caterino sp. n.

http://zoobank.org/DB5D4864-1058-444F-B71E-58F0871998C7

Figures 5 , 10

##### Description.

Body length (exclusive of head and rostrum) 3.2 mm, cuticle almost fully covered with broad, flat scales and scattered short, broad recurved, seta-like scales. Pronotum with lateral margins rounded, margins strongly constricted at about apical one-third such that apical one-third is distinctly tubulate, greatest width at about basal one-third, medially sulcate, median line glabrous, impunctate. Elytra strongly rounded dorsally and laterally, striae distinct, of small punctures; all elytral intervals evenly elevated, slightly rounded, each with single row of broad recurved seta-like scales. Metaventrite medially slightly impressed behind mesoventral cup. Abdomen with ventrite 1 with large median deep glabrous shining pit, area around pit with dense, fine golden inwardly directed scales encircling depression and entering pit along anteriorly along midline such that pit appears somewhat divided. Legs with femora and tibiae fringed with dense erect scales along outer and inner margins, hind tibiae distinctly wider at about midlength. Single specimen likely female, not dissected.

**Figure 10.**
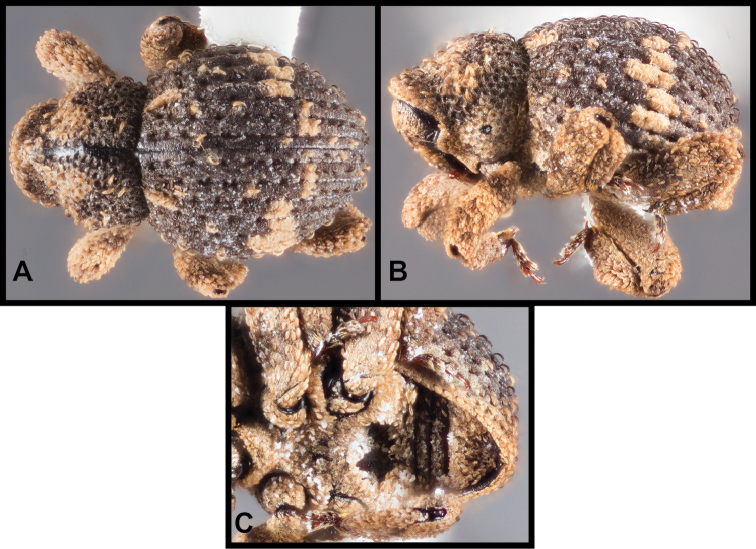
*Eurhoptusrileyi*. **A** Dorsal view **B** Lateral view **C** Ventral view showing impressions on ventrites.

##### Distribution

(Figure 5). This species is known only from a single specimen collected in southern Texas. No records of the species from Mexico are known.

**Figure 11.**
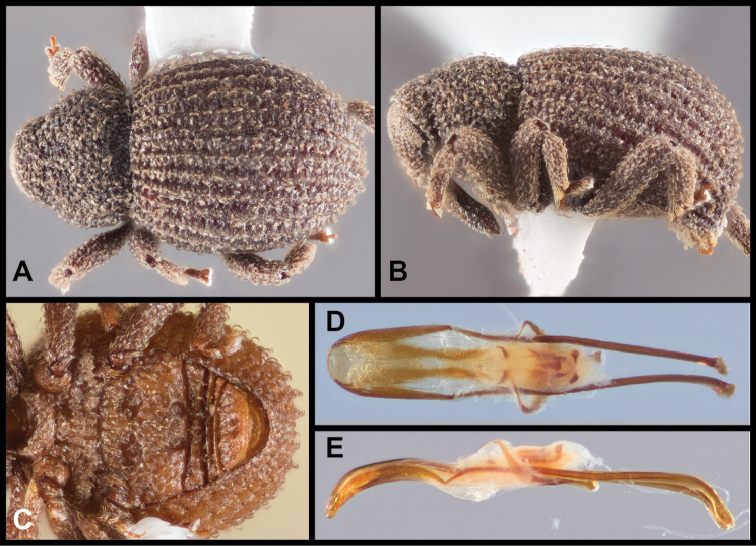
*Eurhoptusoccidentalis*. **A** Dorsal view **B** Lateral view **C** Ventral view showing impressions on ventrites **D** Dorsal view of aedeagus **E** Lateral view of aedeagus.

##### Etymology.

We name this species after Edward G. Riley (retired), former collections manager at the Texas A & M University Insect Collection, College Station, Texas. An invariable word in genitive.

##### Specimens examined.

Holotype female (TAMU), labelled “USA: TEX: Hidalgo Co. / Bentsen R.G.V.S.P. (site 1) / 26.17830°N, 98.38577°W / IX.6–19.2008, FIT-ground / J. King & E. Riley – 0104 / cedar elm forest”, not dissected.

#### Eurhoptus occidentalis

Taxon classificationAnimaliaColeopteraCurculionidae

Anderson & Caterino sp. n.

http://zoobank.org/B207EA8E-A7D6-4D1E-9383-EB12A142D8E4

Figures 5 , 11

##### Description.

Body length (exclusive of head and rostrum) 1.8–2.7 mm, cuticle largely bare, variously covered with dense, short, fine recurved seta-like scales only. Pronotum with lateral margins rounded, margins constricted at about apical one-third such that apical one-third is tubulate, greatest width at about basal one-third, not medially carinate. Elytra strongly rounded dorsally and laterally, striae distinct, punctures moderate in size and depth; all elytral intervals evenly elevated, slightly rounded, each with single row of dense, fine recurved seta-like scales. Metaventrite medially slightly impressed behind mesoventral cup. Abdomen with ventrite 1 with pair of obliquely arranged transversely elongate pits which are separate medially, and with second pair of small rounded pits along posterior margin of ventrite; ventrite 5 with small pair of central, rounded impressions. Legs with femora and tibiae at most fringed with a few erect scales especially along outer margin of tibiae, hind tibiae subequal in width throughout most of length. Aedeagus about as long as one-half length of aedeagal apodemes, in lateral view slightly ventrally curved, in dorsal view with margins gradually convergent from base to apex, apex not produced, broadly rounded. Internal sac with large distinct apical sclerite complex, with median pyriform sclerite, bordered laterally by pair of apically convergent longitudinal bars these each bordered posteriorly by well-defined transverse pyriform sclerite, longitudinal bars not well-defined.

##### Distribution

(Map 2). This species is distributed in west Texas in the Guadalupe and Chisos Mountains. Despite extensive sampling, no specimens are known from New Mexico or Arizona.

##### Etymology.

We name this species “occidentalis” (L., an adjective or participle in the nominative singular, westerly, connected or coming from the west) after its western Texas distribution.

##### Specimens examined.

Holotype male (CMNC), labelled “USA: TEX: Brewster Co. / 29.2611°N, 103.2539°W / BigBend NP, Pine Cyn / 1615 m, ix.6.1988, hardwd / Berlese, R.S. Anderson”, dissected. Paratypes (261): **TEXAS**: Brewster Co.: Big Bend National Park, Pine Canyon, 1615 m (29.2611, -103.2539), R.S. Anderson, 6 Sep 1988, hardwood litter berlese (26, CMNC; 3, CNCI); Brewster Co.: Big Bend National Park, Oak Canyon (29.28, -103.34), R.S. Anderson, 8 Sep 1988, hardwood berlese (1, CUAC); Brewster Co.: Big Bend National Park, Pine Canyon (29.27, -103.24), R.S. Anderson, 6 Sep 1988, hardwood berlese (1, CUAC); Brewster Co.: Big Bend National Park, Window Trail (29.27, -103.32), W. Suter, 29 May 1983, moldy log (1, CUAC); Brewster Co.: Big Bend National Park, Boot Canyon & Emory Peak Trail, 2070 m (29.245, -103.304), R.S. Anderson, 7 Sep 1988, berlese hardwood litter (1, CMNC); Brewster Co.: Big Bend National Park, Boot Spring, 6300’ (29.24, -103.298), S.B. Peck, 7 Aug 1975, oak-maple litter (1, CMNC); Brewster Co.: Big Bend National Park, Cattail Falls, 1310 m (29.273, -103.336), R.S. Anderson, 6 Sep 1988, berlese hardwood litter (14, CMNC; 4, TAMU); Brewster Co.: Big Bend National Park, Cattail Falls (29.273, -103.3356), R.S. Anderson, hardwood berlese (1, CUAC); Brewster Co.: Big Bend National Park, Cattail Falls, (29.273, -103.336), C.E. Carlton, 4 Aug 2000, berlese (10, LSAM); Brewster Co.: Big Bend National Park, below Cattail Falls, (29.275, -103.317), W. Suter, 30 Mar 1983, oak treehole (16, CWOB); Brewster Co.: Big Bend National Park, Cattail Falls, 4300’ (29.273, -103.336), S.B. Peck, 5 Aug 1975, forest litter (10, CMNC; 7, CWOB); Brewster Co.: Big Bend National Park, Green Gulch, 1700m (29.278, -103.284), W. Suter, 1 Jun 1983, litter oak-juniper (1, CMNC); Brewster Co.: Big Bend National Park, Green Gulch, nr. Panther Pass, 1700 m (29.278, -103.284), W. Suter, 1 Jun 1983, litter oak-juniper (1, CWOB); Brewster Co.: Big Bend National Park, 7.4 km. up from Basin (29.27, -103.30), C.E. Carlton, 31 Jul 2000, berlese (6, LSAM); Brewster Co.: Big Bend National Park, Lost Mine Trail, (29.27, -103.28), W. Suter, 6 Jun 1983, litter (1, CWOB); Brewster Co.: Big Bend National Park, Oak Canyon, 1463 m (29.281, -103.336), R.S. Anderson, 8 Sep 1988, berlese hardwood litter (11, CMNC); Brewster Co.: Big Bend National Park, Oak Spring, (29.28, -103.33), W. Suter, 8 Jun 1983, litter under oaks (3, CWOB); Brewster Co.: Big Bend National Park, Pine Canyon, 1615 m (29.2611, -103.2539), R.S. Anderson, 6 Sep 1988, hardwood litter berlese (6, BMNH; 18, CMNC; 6, USNM); Brewster Co.: Big Bend National Park, Pine Canyon, 1830 m (29.267, -103.253), S.B. Peck, 9 Aug 1975, oak maple litter (4, CMNC); Brewster Co.: Big Bend National Park, The Window, 4500’ (29.275, -103.319), S.B. Peck, 2 Aug 1975, (9, CMNC); Brewster Co.: Big Bend National Park, Window Outlet, Rd to Cattail Falls, 1200 m (29.273, -103.34), R.S. Anderson, 6 Sep 1988, berlese hardwood litter (5, CMNC); Brewster Co.: Big Bend National Park, Window Trail, 1370 m (29.275, -103.317), S.B. Peck, 2 Aug 1975, madrone-oak litter (6, ASUIC; 10, CMNC; 11, CWOB; 6, TMMC); Brewster Co.: Big Bend National Park, Window Trail, 1370 m (29.275, -103.317), W. Suter, 29 May 1983 (4, CMNC; 6, CWOB); Brewster Co.: Big Bend National Park, Window Trail, 1370 m (29.275, -103.317), W. Suter, 27 August 1983 (14, CWOB); Brewster Co.: Big Bend National Park, Basin Area, (29.27, -103.30), E.G. Riley, 20–21 July 2002, berlese oak ravine litter (10, EGRC; 23, TAMU);) ; Brewster Co.: Big Bend National Park, Emory Peak Trail (29.26, -103.30), C.E. Carlton, 4 Aug 2000, berlese (2, LSAM); Culberson Co.: Guadalupe Mountains National Park, McKittrick Canyon (31.98, -104.78), P.W. Kovarik, 4 Sep 1986, leaf litter (2, CMNC).

#### Eurhoptus aenigmaticus

Taxon classificationAnimaliaColeopteraCurculionidae

Anderson & Caterino sp. n.

http://zoobank.org/53E13EA1-3692-4194-A23B-2BF35ADE5DCF

Figures 12 , 13

Eurhoptus sordidus (part); O’Brien and Wibmer 1982: 136; Downie and Arnett 1996: 1581.

##### Description.

Body length (exclusive of head and rostrum) 2.2–2.5 mm, cuticle largely bare, variously covered with short, fine recurved seta-like scales only, although often encrusted and coated with a fine film. Pronotum with lateral margins rounded, margins constricted at about apical one-third such that apical one-third is tubulate, greatest width at about basal one-third, medially carinate (most) or not (few). Elytra strongly rounded dorsally and laterally, striae distinct, punctures large in size, deep; all elytral intervals evenly elevated, slightly rounded, each with single row of fine recurved seta-like scales. Metaventrite medially deeply impressed behind mesoventral cup. Abdomen with ventrite 1 with pair of rounded pits which are continuous medially, area around pits with inwardly directed scales. Legs with femora and tibiae at most fringed with a few erect scales especially along outer margin of tibiae, hind tibiae subequal in width throughout most of length. Aedeagus slightly longer than one-half length of aedeagal apodemes, in lateral view very slightly ventrally curved, in dorsal view with margins gradually tapered towards very blunt, truncate apex, apex not produced medially. Internal sac with large distinct apical sclerite complex, with median pyriform sclerite, bordered laterally by pair of inwardly arcuate longitudinal bars these each bordered posteriorly by an elongate oblique sclerite.

**Figure 12.**
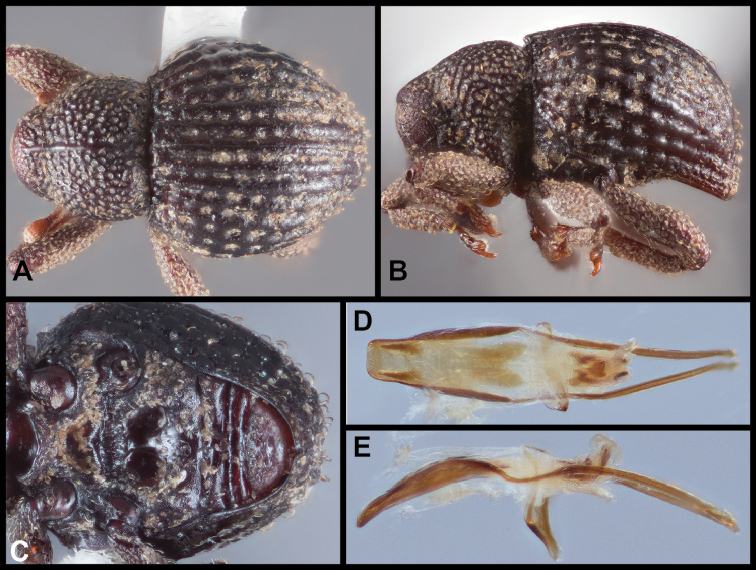
*Eurhoptusaenigmaticus*. **A** Dorsal view **B** Lateral view **C** Ventral view showing impressions on ventrites **D** Dorsal view of aedeagus **E** Lateral view of aedeagus.

##### Distribution

**Figure 13.**
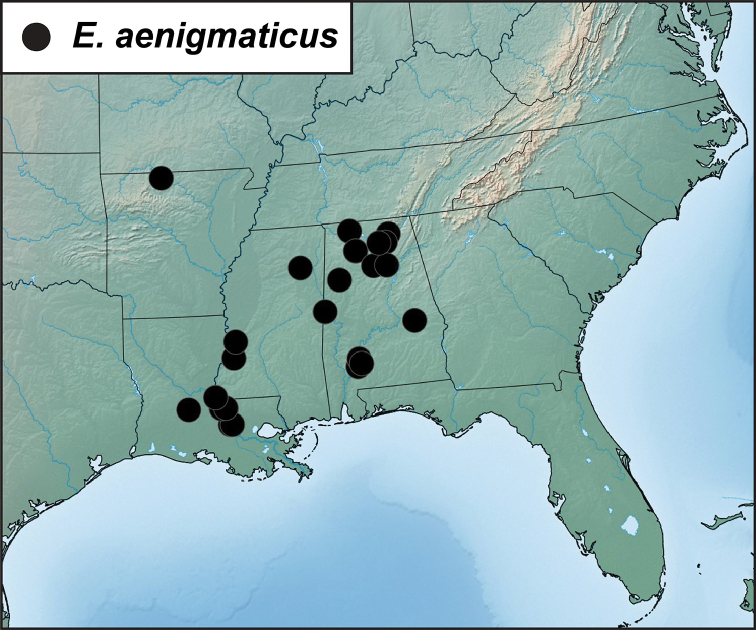
Records of *Eurhoptusaenigmaticus*.

(Figure 13). This species is distributed in the southern coastal plain of the U.S.A. from Alabama and Florida west to Louisiana. We have been able to sequence KKV, CAD, and COI from some individuals from Alabama. In COI these are rather divergent, though the other two genes show minimal difference from *Eurhoptuscurtus*. Further effort to obtain sequences from these and other populations of *E.aenigmaticus* will help support its placement relative to *E.sordidus* and *E.curtus*.

##### Etymology.

We name this species ‘aenigmaticus’ (L., an adjective or participle in the nominative singular, enigma, puzzle or riddle) after encountering difficulty in separating it from closely related species of the genus.

##### Specimens examined.

Holotype male (CMNC), labelled “AL: Winston Co.: Bankhead NF / Brushy Ck Lake CG, N34.297° / W87.2697°cols. CEC, MLF, JSP, AT / 3.Wink216h3/4”, dissected. Extracted SSM-478. Paratypes (163): **ALABAMA**: Blount Co.: Blount Springs (33.9, -86.8), T. King, 31 Oct 1982, oak tree hole (1, UAAM); Blount Co.: Highland Lake (33.88, -86.42), T. King, 6 May 2009, litter (2, CWOB); Blount Co.: Highland Lake (33.88, -86.42), T. King, 20 Mar 2010, litter (1, CWOB); Cleburne Co.: Cheaha State Park (32.493, -85.809), R. Brown; J.A. McGown, 16 May 1998, berlese magnolia leaf litter (2, MEM); Jackson Co.: 3.2 km. N. Scottsboro (34.75, -86.04), 16 May 1972, S. & J. Peck (1, CNCI); Jackson Co.: Woodville (34.627, -86.274), S. Peck, 12 Jul 1965, leaf litter (2, CWOB); Lauderdale Co.: Joe Wheeler State Park (34.80, -87.33), J.A. McGown, 27 May 2004, soil humus at base *Fagusgrandofilia* (1, MEM); Joe Wheeler State Park (34.80, -87.33), J.A. McGown, 27 May 2004, litter under rotten log (1, MEM); Joe Wheeler State Park (34.786, -87.388), J.A. McGown, 26 May 2004, mixed litter (1, MEM); Marshall Co.: 1.6 km. N. Guntersville Dam (34.43, -86.39), S. Peck, 11 Jun 1967, litter (1, CWOB); Monroe Co.: Haines Island Reserve (31.724, -87.470), V. Behan, 17 Mar 1994, mixed litter (1, MEM); Monroe Co.: Haines Island Park (31.723, -87.469), C.E. Carlton, 31 May 1995, beech/magnolia litter (3, LSAM); Monroe Co.: Haines Island Park (31.723, -87.469), C.E. Carlton, 26 May 1995, beech/magnolia litter (2, CMNC; 4, LSAM); Monroe Co.: 1.6 km.km. S. Claiborne Dam (31.54, -87.52), A. Tishechkin, 16 Sep 2005, litter (1, CWOB); Monroe Co.: 1.6 km. S. Claiborne Dam (31.54, -87.52), C.E. Carlton, 31 May 1995, litter (2, LSAM); Monroe Co.: Big Flat Creek (31.54, -87.52), A. Tishechkin, 16 Sep 2005, litter (1, CWOB); Morgan Co.: 5.63 km. S.E. Fayette (31.608, -87.414), C.E. Carlton, 27 May 1995, litter (1, LSAM); Morgan Co.: 5.63 km. S.E. Fayette (31.608, -87.414), S.B. Peck, 21 May 1972, litter (2, CMNC); Morgan Co.: Newsom Sinks, Turtle Cave area (34.43, -86.58), A. Cline & J. Skeens, 26 Nov 2004, litter (2, LSAM); Sumter Co.: Highway 17 at Noxubee River (32.918, -88.298), J.A. McGown, 27 May 2004, under dead *Quercusnigra*, floodplain (3, MEM). **LOUISIANA**: East Baton Rouge Par.: Place Du Plantier Apartments (30.4, -91.1546), E.G. Riley, 30 Dec 1993, berlese leaf litter (4, ASUIC; 2, BMNH; 16, CMNC; 2, CNCI; 4, LSAM; 22, TAMU; 2, USNM); East Baton Rouge Par.: Place Du Plantier Apartments (30.4, -91.1546), E.G. Riley, 27 Nov 1993, berlese leaf litter (20, EGRC; 11, TAMU); Evangeline Par.: Chicot State Park (30.795, -92.279), A. Tishechkin , 19 Feb 2004, berlese (4, CMNC); West Feliciana Par.: Feliciana Preserve (30.78, -91.25), J.L. Fassbender, 21 Apr 1999, sifting litter mixed pine hardwood forest (2, CMNC; 1, LSAM); West Feliciana Par.: D.P. Cabin (30.795, -91.256), J.L. Johnson, 21 Dec 1995, berlese (3, CWOB); West Feliciana Par.: Tunica Hills WMA, Magnolia Glen Trail (30.79. -91.38), D. Pashley, 15 Jul 1995 (1, LSAM); West Feliciana Par.: Tunica Hills WMA (30.79. -91.38), D. Pashley, 24 May 1995 (1, LSAM); West Feliciana Par.: Tunica Hills WMA, nr. St. Francisville (30.79. -91.38), J. & T. Fassbender, 23 Sep 1999, lower ravine berlese (10, CWOB). **MISSISSIPPI**: Chickasaw Co.: Natchez Trace Parkway mile 241.1 (34.01, -88.90), J.A. McGown; J.G. Hill, 21 Jul 2003, mixed litter (1, MEM); Claiborne Co.: Natchez Trace Parkway (31.97, -90.956), J.A. McGown; J.G. Hill, 8 Jul 2005, mixed litter (4, MEM); Warren Co.: Vicksburg, Waterways Experiment Station (32.355, -90.878), P.W. Kovarik, 23. Mar 1998, beech magnolia litter (2, CMNC; 14, CWOB); Wilkinson Co.: Clark Creek Natural Area (31.058, -91.521), A. Tishechkin, 2 Dec 2005, forest litter (5, CWOB).

Excluded from type series: **ARKANSAS**: Marion Co.: 7 mi E County line on Hwy 14 (36.36, -92.8), R. Chenowith, 27 Sep 1977, mixed hardwood litter (1, ASUIC).

#### Eurhoptus cariniventris

Taxon classificationAnimaliaColeopteraCurculionidae

Anderson & Caterino sp. n.

http://zoobank.org/59AF7223-87AA-4BAF-B4F2-0C9DBB65CC53

Figures 9 , 14

Eurhoptus pyriformis (part); Blatchley and Leng 1916: 497; Kissinger 1964: 64; Papp 1979: 164; O’Brien and Wibmer 1982: 136; Downie and Arnett 1996: 1581; Alonso-Zarazaga and Lyal 1999: 131; Anderson 2002: 765.

##### Description.

Body length (exclusive of head and rostrum) 1.8–2.4 mm, cuticle largely bare, variously covered with short, fine recurved seta-like scales, most specimens lacking other scales but some with head and base of rostrum scaly and with a few broad, flat pale scales variously arranged on elytra in apical one-half and at bases of intervals 2–4. Pronotum with lateral margins straight, margins tapered more or less evenly from base to apex with greatest width at base, medially carinate or not. Elytra strongly rounded dorsally and laterally, almost globular, striae distinct, of very large punctures, especially in posthumeral region; all elytral intervals evenly elevated, slightly rounded, each with single row of fine recurved seta-like scales. Metaventrite medially distinctly carinate behind mesoventral cup. Abdomen with ventrite 1 with large median deep glabrous shining pit, area around pit with dense, fine golden inwardly directed scales encircling depression. Legs with femora and tibiae fringed with erect scales along outer margin of tibiae, hind tibiae subequal in width throughout most of length to slightly wider at basal one-third. Aedeagus about as long as one third length of aedeagal apodemes, in lateral view almost straight, in dorsal view with margins subparallel but strongly tapered towards apex at about midlength, apex produced medially as fine acuminate point. Internal sac with distinct apical sclerite complex of pair of longitudinal parallel bars.

**Figure 14.**
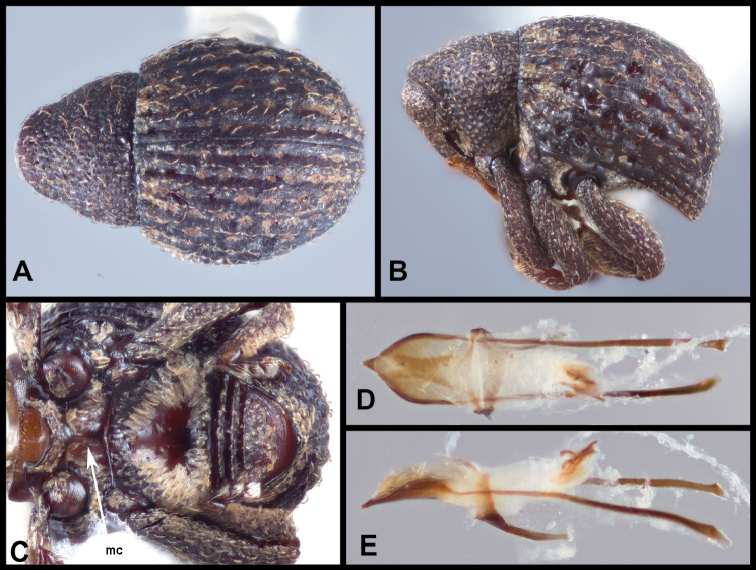
*Eurhoptuscariniventris*. **A** Dorsal view **B** Lateral view **C** Ventral view showing impressions on ventrites and metaventral carina (arrow, mc) **D** Dorsal view of aedeagus **E** Lateral view of aedeagus.

##### Distribution

(Figure 9). This species is distributed throughout central Texas and appears to be disjunct to the panhandle of Florida and adjacent Alabama and Georgia. All published Texas records of *E.pyriformis* are this species.

##### Etymology.

We name this species “cariniventris” (L., an adjective or participle in the nominative singular, carina, keeled, and ventris, belly) after the distinctly carinate metaventrite posterior to the mesoventral cup.

##### Variation.

This species appears to have two disjunct portions of its range; central Texas and Florida panhandle and adjacent Alabama into eastern Georgia. We know of no specimens from the intervening areas. Specimens from Florida/Alabama have larger, deeper elytral punctures (especially in the posthumeral area) but have similar male genitalia and do not differ in other structural characters. At present, we consider these eastern specimens to be conspecific with the Texas specimens but have excluded them from the type series.

##### Specimens examined.

Holotype male (CMNC), labelled “TEX: Bandera Co. / Lost Maples St. Nat. Area / 28.IV.-2.V.87 R. Anderson / Berlese leaf/log litter”, dissected. Paratypes (312): **TEXAS**: Bandera Co.: Lost Maples State Natural Area (29.82, -99.57), R.S. Anderson, 28 Apr-2 May 1987, leaf/log litter (42, CMNC; 5, CNCI; 1, CUAC; 5, CWOB; 1, UCFC); Bandera Co.: Lost Maples State Natural Area (29.82, -99.57), R.S. Anderson, 28–30 Apr 1988, leaf litter (42, CMNC; 1, CUAC; 5, CWOB); Bandera Co.: Lost Maples State Natural Area (29.82, -99.57), C.E. Carlton, 19 May 2005, forest litter (2, LSAM); Bandera Co.: Lost Maples State Natural Area, East Trail to pond (29.82, -99.58), C.W. O’Brien, 9 Mar 2001, sifted mixed hardwood litter (2, ASUIC); Bandera Co.: Lost Maples State Natural Area, Maple Trail (29.82, -99.57), C.W. O’Brien & Gages, 10 Mar 2001, sifted mixed hardwood litter (1, ASUIC); Bandera Co.: Lost Maples State Natural Area (29.82, -99.57), E.G. Riley, 24 Apr 2005, berlese maple forest litter (1, EGRC); Bandera Co.: Lost Maples State Natural Area (29.82, -99.57), E.G. Riley, 11 May 1997, forest litter (2, EGRC); Bandera Co.: Lost Maples State Natural Area (29.82, -99.57), E.G. Riley, 27 Mar 1999, forest litter (1, EGRC); Bandera Co.: Lost Maples State Natural Area (29.82, -99.57), P.W. Kovarik, 21 Apr 1986, leaf litter (1, TAMU); Bandera Co.: Lost Maples State Natural Area (29.82, -99.57), P.W. Kovarik, 26 Apr 1986, leaf litter (12, TAMU); Bandera Co.: Lost Maples State Natural Area (29.82, -99.57), P.W. Kovarik, 10 May 1986, leaf litter (3, TAMU); Bastrop Co.: Buescher State Park (30.041, -97.162), R.S. Anderson, 3 May 1988, leaf litter berlese (15, CMNC); Bastrop Co.: Buescher State Park (30.041, -97.162), R.S. Anderson, 3 May 1988, leaf litter (1, CUAC); Blanco Co.: Pedernales Falls State Park, Nature Trail (30.306, -98.256), R.S. Anderson, 3 May 1988, oak/elm/juniper litter (10, CMNC); Colorado Co.: 2 mi S Columbus (29.6, -96.5), C.W. O’Brien & G.J. Wibmer, 12 Dec 1984, sifted hardwood litter (1, ASUIC); Colorado Co.: Columbus (29.7065, -96.5353), R.S. Anderson, 17 May 1987, riparian ravine hardwood litter (1, CUAC); Colorado Co.: Columbus (29.7065, -96.5353), R.S. Anderson, 1 Feb 1989, riparian ravine hardwood litter (1, CUAC); Colorado Co.: Columbus (29.7072, -96.5344), R.S. Anderson, 1 Feb 1988, riparian ravine litter (25, CMNC); Colorado Co.: Columbus (29.7072, -96.5344), R.S. Anderson, 22 Mar 1988, riparian ravine litter (8, CMNC); Colorado Co.: Columbus (29.7072, -96.5344), R.S. Anderson, 4 Nov 1988, riparian ravine litter (33, CMNC); Colorado Co.: Columbus (29.7072, -96.5344), R.S. Anderson, 21 Feb 1989, riparian ravine litter (10 BMNH; 31, CMNC; 1, CUAC; 10 CWOB; 10, TAMU; 5, TMMC; 10 USNM); Colorado Co.: Columbus (29.7065, -96.5353), C.V. Riley, 24 Aug 1929 (1, CWOB); Fort Bend Co.: Brazos Bend State Park (29.37, -95.63), B. Raber & E.G. Riley, 15 Jun 2000, FIT (1, EGRC); Travis Co.: Austin, Mayfield Park (30.3126, -97.771), R. Jones, 15 Apr 1989, leaf litter (10, CMNC); Travis Co.: Austin, Brackenridge Field Lab (30.284, -97.778), E.G. Riley, 10 Feb 1996, berlese rotten pecan log (1, EGRC).

Excluded from type series: **ALABAMA**: Dale Co.: Fort Rucker Military Reserve (31.29, -85.72), R. Turnbow, 31 Mar 1993, leaf litter (1, CMNC); Dale Co.: Fort Rucker Military Reserve (31.29, -85.72), R. Turnbow, 27 Sep 1991, leaf litter (1, CMNC); Dale Co.: Fort Rucker (31.349, -85.711), R.H. Turnbow, Jr., 27 Oct 2001 (1, CWOB). **FLORIDA**: Gadsden Co.: 4 mi SE Havana, junction Co. Rd. 153 & Ochlokonee River (30.58, -84.36), C.W. O’Brien & G.B. Marshall, 23 Mar 2001, sifted hardwood litter (1, ASUIC; 3, CWOB); Gilchrist Co.: Hart Springs (29.675, -82.951), L.R. Davis Jr., 23 Apr 1993 (1, CWOB); Jackson Co.: Florida Caverns State Park (30.812, -85.225), P.E. Skelley, 8 Apr 1994, leaf litter around rock outcroppings, cave area (4, CMNC; 2, CWOB; 3 FSCA); Liberty Co.: TNC Apalachicola Bluffs & Ravines Preserve, Travelers Tract (30.46, -84.98), P.E. Skelley, 23 Mar 1996, leaf litter (3, ASUIC); Liberty Co.: Okaloosa Co.: Milligan (30.752, -86.64), G.B. Marshall, 21 Apr 1977 (2, CWOB); Torreya State Park (30.56, -84.96), R. Turnbow, 25 Jun-8 Jul 1989, pitfall (2, FSCA); Liberty Co.: Torreya State Park (30.56, -84.96), C.W. O’Brien & G.B. Marshall, 23 Mar 2001, berlese beech litter (2, CWOB). **GEORGIA**: Tatnall Co.: Big Hammock Natural Area, 19.3 km. S.W. Glennville (31.863, -82.058), C.W. O’Brien & P. Kovarik, 20 Mar 1999, live oak, mixed hardwood litter (1, CWOB).

### Key to species of *Eurhoptus* LeConte in America north of Mexico

**Table:** 

1	Body surface almost fully densely covered with broad flat scales, elytra with an irregular oblique band of lighter scales across elytra in apical one-half (Figures 8, 10). Legs with femora and tibiae fringed with dense erect scales on both inner and outer margins, hind tibiae with width distinctly greatest at basal one-third of length or at midlength	**2**
–	Body surface lacking broad flat scales, or with a few broad, flat scales arranged in an irregular oblique band across elytra in apical one-half and at bases of intervals 3–5. Legs with tibiae at most fringed with a few erect scales especially along outer margin, hind tibiae subequal in width throughout most of length (except some *E.cariniventris* which have tibiae fringed along outer margin with dense scales, hind tibia slightly wider at basal one-third)	**3**
2	Pronotum with broad, glabrous median line extended from apical one-quarter to base (Figure 10A), line recessed in a broad furrow between dorsal swellings on disc. Elytra with broad flat scales dense, imbricate, matte; intervals each with single row of rather broad, fully arched seta-like scales. Abdomen with large median pit on ventrite 1 lacking erect scales within pit (Figure 10C). (Hidalgo County, Texas)	*** E. rileyi ***
–	Pronotum lacking median line or with narrow median glabrous carina of various length (Figure 8A), pronotal disc uniformly rounded, lacking any dorsal swellings. Elytra with scales dense, imbricate, shining (may be matted); intervals each with single row of incompletely arched to appressed, narrow seta-like scales. Abdomen with large median pit on ventrite 1 with numerous erect scales within pit (Figure 8C)	*** E. imbricatus ***
3	Pronotum with lateral margins more or less uniformly convergent from base to apex (e.g. Figure 2A), greatest width at base. Abdomen with ventrite 1 with single large cavernous, shining median pit (Figure 2E)	**4**
–	Pronotum with lateral margins rounded from apex to base (e.g. Figure 4A), greatest width in front of base. Abdomen with ventrite 1 with paired separate small round shallow pits (Figures 4C or 11C) to larger transversely elongate-oval deep pits that are confluent medially (Figure 6C)	**5**
4	Metaventral midline raised and carinate (Figure 14C). Elytra with scales of intervals fine, narrow. Many specimens with elytral punctures large and pit-like in area behind humeri (Figure 14B). Aedeagus as in Figures 14D–E	*** E. cariniventris ***
–	Metaventral midline impressed, not raised. Elytra with scales of intervals slightly broader but narrow. Most specimens with elytral punctures slightly larger in area behind humeri but not large and pit-like (Figure 2B). Aedeagus as in Figures 2F–H	*** E. pyriformis ***
5	Abdomen with ventrite 1 with four distinct impressions (2 anteriorly, 2 posteriorly; Figure 11C). Aedeagus as in Figures 11D–E	*** E. occidentalis ***
–	Abdomen with ventrite 1 with two distinct impressions (not, or variously confluent medially)	**6**
6	Impressions of abdominal ventrite 1 separate (Figure 4C). Pronotum with anterior constriction strong, lateral margins strongly sinuate; Aedeagus as in Figures 4D–E	*** E. sordidus ***
–	Impressions of abdominal ventrite 1 variously confluent. Pronotum with anterior constriction weak, lateral margins slightly sinuate	**7**
7	Aedeagus as in Figures 12D–E. Pronotum usually with distinct median carina. Abdomen with pits of ventrite 1 smaller, rounder (Figure 12C)	*** E. aenigmaticus ***
–	Aedeagus as in Figures 6D–E. Pronotum usually lacking distinct median carina. Abdomen with pits of ventrite 1 larger, more obliquely oval (Figure 6C)	*** E. curtus ***

## Supplementary Material

XML Treatment for Eurhoptus

XML Treatment for Eurhoptus pyriformis

XML Treatment for Eurhoptus sordidus

XML Treatment for Eurhoptus curtus

XML Treatment for Eurhoptus imbricatus

XML Treatment for Eurhoptus rileyi

XML Treatment for Eurhoptus occidentalis

XML Treatment for Eurhoptus aenigmaticus

XML Treatment for Eurhoptus cariniventris

## Supplementary materials

Supplementary material 1 Table S1. Full data and GenBank accession numbers for all individuals and genes sequenced Data type: molecular data This is an open access article distributed under the terms of the Creative Commons Attribution License (CC BY 4.0), which permits unrestricted use, distribution, and reproduction in any medium, provided the original author and source are credited.Robert S. Anderson, Michael S. Caterino
